# Physico‐Chemical Properties of Magnetic Dicationic Ionic Liquids with Tetrahaloferrate Anions

**DOI:** 10.1002/open.202200229

**Published:** 2023-01-04

**Authors:** Anham Zafar, Imtiaz‐ud‐Din, Robert G. Palgrave, Haji Muhammad, Sammer Yousuf, Tim Evans

**Affiliations:** ^1^ Chemistry Department University College London 20 Gordon Street London WC1E 0AJ UK; ^2^ Department of Chemistry Quaid-i-Azam University Islamabad 453208 Pakistan; ^3^ Department of Chemistry Federal Urdu University of Arts, Sciences and Technology Karachi 75300 Pakistan; ^4^ H.E.J. Research Institute of Chemistry International Center for Chemical and Biological Sciences University of Karachi Karachi 75270 Pakistan

**Keywords:** dicationic ionic liquids, iron, magnetic susceptibility, physico-chemical properties, X-ray crystal analysis

## Abstract

A series of imidazolium‐based symmetrical and asymmetrical dicationic ionic liquids (DcILs) with alkyl spacers of different length and with [FeCl_3_Br]^−^ as counter ion have been synthesized. The synthesized DcILs are characterized by using FTIR and Raman spectroscopy as well as mass spectrometry, along with single‐crystal XRD analysis. Physicochemical properties such as solubility, thermal stability and magnetic susceptibility are also measured. These compounds show low melting points, good solubility in water and organic solvents, thermal stability, and paramagnetism. The products of molar susceptibility and temperature (χ_mol_⋅T) for the synthesized DcILs have been found between 4.05 to 4.79 emu mol^−1^ K Oe^−1^ and effective magnetic moment values have also been determined to be compared to that expected from the spin‐only approximation.

## Graphical abstract

**A series of dicationic ionic liquids** based on symmetrically and asymmetrically substituted imidazolium units combined with magnetic tetrahaloferrate anions have been synthesized and their magnetic and physical properties have been measured.

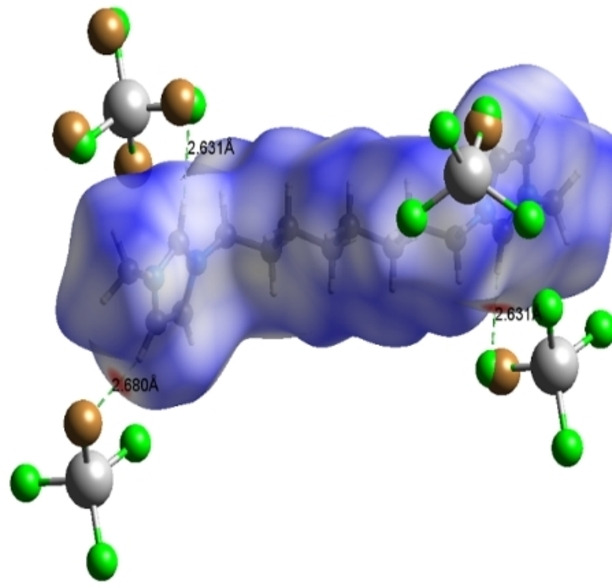

## Introduction

Ionic liquids (ILs) have been an interesting topic of scientific research in recent years and their application in various fields is constantly increasing.^[1]^ New advances in air‐and moisture‐stable room‐temperature imidazolium‐based ionic liquids^[2]^ have driven the boundaries of their applications beyond solvents,^[3]^ synthetic chemistry,^[4]^ lubricants,^[5]^ catalysis,^[6]^ and electrochemistry^[7]^ to a new range of applications.^[8]^

The low melting point of ILs is due to hinderance of the ionic interaction between the anion and cation and prevention of ordered closed packing within a crystal lattice: such effects are caused by large, asymmetric cations and anions.^[9]^ Developments in IL design have not only extended the chemical space but also permitted the extraordinary flexibility for a variety of anions and cations to the ILs with tuneable properties for fundamental studies and practical applications.[^10^, ^11^]

Dicationic ionic liquids (DcILs) are subclass of ILs which contain two cationic units linked by a spacer chain, forming a single dication. The dication can either be symmetrical (with the same cation group at each end of the spacer) or asymmetrical, and the spacer chain can be flexible or rigid.^[12]^ DcILs provide additional options for control of properties over that offered by simple mono cationic ILs^[13]^ and also possess promising physical and chemical properties like low density,^[14]^ high viscosity,^[15]^ thermal stability,^[16]^ attractive refractive indeces,^[17]^ and controllable glass transition temperature^[18]^ as well as good solvation properties.^[19]^ They provide more opportunities to meet task specific requirements by changing the chain length of the spacer group,^[20]^ in addition to the identity of the cation end groups and the anion. For example, their melting points and other physicochemical properties can also be manipulated greatly by forming either symmetric^[21]^ or asymmetric cations.^[22]^

Imidazolium‐based DcILs, due to many applicable structural variations and improved physicochemical properties,^[23]^ have gained significant importance in the field of organic synthesis,^[24]^ electro‐membrane extraction,^[25]^ catalysis,^[26]^ and lithium ion batteries^[27]^ while the DcILs with transition‐metal‐containing anions exhibit strong magnetic response,^[28]^ offer new supramolecular architecture and potential applications arising from their magnetic properties (magnetic fluids,^[29]^ transport and separation of magnetic materials^[30]^), and absorbent liquids to lubrication^[31]^ and catalysis.^[32]^ Here, we describe magnetic dicationic ionic liquids, consisting of a high‐spin d^5^‐iron(III) system in the form of [FeCl_3_Br]^−^ as paramagnetic counter anion which is also responsible for lowering the melting point while still permitting the tunability of many physicochemical properties with the retention of magnetic responsivity.^[33]^

Keeping these aspects in view and in continuation to our previous work,[^34^, ^35^, ^36^] here, imidazolium‐based symmetrical and asymmetrical DcILs linked with different alkyl chains (spacer groups), and [FeCl_3_Br]^−^ as counter ion, have been synthesized. The characterization of these DcILs has been carried out through ^1^H, ^13^C NMR, FTIR, and Raman spectroscopy along with mass spectrometry, thermogravimetry and single‐crystal XRD analyses. Their magnetic properties have also been investigated for their possible uses in applied fields such as surfactants,^[37]^ CO_2_ separation,^[38]^ analytical extraction^[39]^ and extractive desulfurization.^[40]^

## Experimental Section

### Materials and Instrumentation

All regents and solvents of 99 % or greater purity were purchased from Sigma‐Aldrich Chemical Co. and used as received. Melting points were determined using an Electrothermal IA9300 apparatus and are uncorrected. Infrared (IR) spectra were recorded as neat using a Bruker Alpha spectrometer. The ^1^H and ^13^C NMR spectra were taken in D_2_O solvent and recorded on Bruker Avance III 400 spectrometer (^1^H: 400 MHz). Electrospray analyses were performed by mass spectrometry facility using the Vanquisher LC system connected to the Orbitrap Exactive Plus mass spectrometer operating in positive ionization mode with an HESI ion source. Raman spectra were acquired using a Renishaw in Via Raman microscope with a 50× objective in a back‐scattering configuration. The excitation wavelength was 785 nm (130 mW, 10 %) and the acquisition time was 10 s. The laser spot size was around 1 μm^2^. A Netzsch Jupiter TGA system was used for thermogravimetric analysis at a heating rate of 10 °C/min in the range of 20 to 800 °C. A PerkinElmer Fourier Transform Lambda 950 UV‐vis spectrophotometer equipped with an integrating sphere was used to measure the optical diffuse reflectance spectra between 200 to 800 nm with a step size of 1 nm .The magnetic susceptibility was measured using an Evans balance. To measure the magnetic susceptibility as a function of temperature, an AC magnetic susceptometer was used.

### Synthesis

Symmetric and asymmetric dicationic ionic liquids were synthesized using a previously reported procedure and are shown in Table 1.[^13^, ^38^, ^41^] One molar equivalent of 1,n‐dibromoalkane and two molar equivalents of the required imidazoles were mixed under magnetic stirring and heated at reflux for 12 h. A thick white gel‐like material was formed within an hour. After 12 h, this crude dibromide salt was washed with ethyl acetate to remove the excess of starting materials followed by vacuum drying at 100 °C for 24 h that ensued the dication with bromide anions. A metathesis reaction was carried out to prepare the desired dicationic ionic liquids. Stochiometric amounts of the respective synthesized dibromide salt and FeCl_3_ were stirred in absolute ethanol for 8 h at room temperature. Dicationic ionic liquids with the [FeCl_3_Br]^−^ anion were obtained in excellent yields and were subsequently washed with diethyl ether and n‐hexane to obtain pure products.

***1,1’‐(Octane‐1,8‐diyl)‐bis(3‐methylimidazolium) dibromide (I***_***1***_ ***)***: ^1^H NMR (400 MHz, D_2_O, ppm), δ: 8.71 (s, 2H, N−CH−N), 7.48 (m, 4H, Ar−H), 4.19 (t, ^3^ *J*_HH_=8 Hz, 4H, N−CH_2_), 3.89 (s, 6H, N−CH_3_), 1.86 (m, 4H, −CH_2_), 1.32 (m, 8H, −CH_2_); ^13^C NMR (100 MHz, D_2_O, ppm) δ: 124.01 (N−CH−N), 122.63 (Ar−C), 49.87 (N−CH_2_), 36.12 (N− CH_3_), 29.57, 25.38 (−CH_2_).

***1,1’‐(Pentane‐1,5‐diyl)‐bis(3‐methylimidazolium) dibromide (I***_***2***_ ***)***: ^1^H NMR (400 MHz, D_2_O, ppm), δ: 9.34 (s, 2H, N−CH−N), 7.86 (m, 4H, Ar−H), 4.20 (t, ^3^ *J*_HH_=8 Hz, 4H, N−CH_2_),3.87 (s, 6H, N−CH_3_), 1.83 (m, 4H), 1.22 (m, 2H). ^13^C NMR (100 MHz, D_2_O, ppm) δ: 139.81 (N−CH−N), 126.83 (Ar−C), 51.59 (N−CH_2_), 39.04 (N−CH_3_), 31.87, 25.19 (−CH_2_).

***1,1’‐(Ethane‐1,2‐diyl)‐bis(3‐methylimidazolium) dibromide (I***_***3***_ ***)***: ^1^H NMR (400 MHz, D_2_O, ppm), δ: 8.82 (s, 2H, N−CH−N), 7.55 (m, 4H, Ar−H), 3.94 (m, 10H, N−CH_2_). ^13^C NMR (100 MHz, D_2_O, ppm) δ: 125. 11 (N−CH−N), 122.66 (Ar−C), 49.28 (N−CH_2_), 36.52 (N−CH_3_).

***1,1’‐(Octane‐1,8‐diyl)‐bis(3‐butylimidazolium) dibromide (I***_***4***_ ***)***: ^1^H NMR (400 MHz, D_2_O, ppm), δ: 8.77 (s, 2H, N−CH−N), 7.49 (m, 4H, Ar−H), 4.20 (m, 8H, N−CH_2_), 1.85 (m, 8H, −CH_2_), 1.31 (m, 12H, −CH_2_), 0.92 (t, ^3^ *J*_HH_=8 Hz, 6H, −CH_3_); ^13^C NMR (100 MHz, D_2_O, ppm) δ: 135.51(N−CH−N), 122.89 (Ar−C), 50.14 (N−CH_2_), 31.86, 28.57, 25.88, 19.35 (−CH_2_), 13.22 (−CH_3_).

***1,1’‐(Pentane‐1,5‐diyl)‐bis(3‐butylimidazolium) dibromide (I***_***5***_ ***)***: ^1^H NMR (400 MHz, D_2_O, ppm), δ: 7.76 (s, 2H, N−CH−N), 7.51 (m, 4H, Ar−H), 4.21 (m, 8H, N−CH_2_), 1.86 (m, 8H, −CH_2_), 1.32 (m, 6H, −CH_2_), 0.93 (t, ^3^ *J*_HH_=8 Hz, 6H, −CH_3_). ^13^C NMR (100 MHz, D_2_O, ppm) δ: 122.93 (N−CH−N), 122.77 (Ar−C), 49.88 (N−CH_2_), 31.75, 29.19, 22.79, 19.41 (−CH_2_), 13.14 (−CH_3_).

***1,1’‐(Ethane‐1,2‐diyl)‐bis(3‐butylimidazolium) dibromide (I***_***6***_ ***)***: ^1^H NMR (400 MHz, D_2_O, ppm), δ: 8.83 (s, 2H, N−CH−N), 7.62 (m, 4H, Ar−H), 4.21 (m, 8H, N−CH_2_), 1.83 (m, 4H), 1.26 (m, 4H), 0.92 (t, ^3^ *J*_HH_=16 Hz, 6H, −CH_3_). ^13^C NMR (100 MHz, D_2_O, ppm) δ: 124.07 (N−CH−N), 122.75 (Ar−C), 50.20 (N−CH_2_), 31. 67, 19.24 (−CH_2_), 13.08 (−CH_3_).

***1‐(3‐Methylimidazolium‐yl‐octyl)‐3‐butylimidazolium dibromide (I***_***7***_ ***)***: ^1^H NMR(400 MHz, D_2_O, ppm), δ: 8.79 (m, 2H, N−CH−N), 7.49 (m, 4H, Ar−H), 4.20 (t, ^3^ *J*_HH_=8 Hz, 6H, N−CH_2_), 3.89 (s, 3H, N−CH_3_), 1.86 (m, 6H), 1.31 (m, 10H, −CH_2_), 0.92 (t, ^3^ *J*_HH_=8 Hz, 3H, −CH_3_); ^13^C NMR(100 MHz, D_2_O, ppm) δ: 136.21 (N−CH−N), 124.03 (Ar−C), 50.01 (N−CH_2_), 36.10 (N−CH_3_), 31.86, 29.81, 28.57, 19.37 (−CH_2_), 13.24 (CH_3_).

***1‐(3‐Methylimidazolium‐yl‐pentyl)‐3‐butylimidazolium dibromide (I***_***8***_ ***)***: ^1^H NMR (400 MHz, D_2_O, ppm), δ: 7.49(m, 6H, Ar−H), 4.20 (t, ^3^ *J*_HH_=8 Hz, 6H, N−CH_2_), 3.89 (s, 3H, N−CH_3_), 1.92 (m, 6H, −CH_2_), 1.31 (m, 4H, −CH_2_), 0.92 (t, ^3^ *J*_HH_=8 Hz, 3H, −CH_3_). ^13^C NMR (100 MHz, D_2_O, ppm) δ: 124.08(N−CH−N), 122.93 (Ar−C), 49.86 (N−CH_2_), 36.17 (N−CH_3_), 31.74, 29.24, 22.73, 19.28 (−CH_2_), 13.13 (−CH_3_).

***1‐(3‐Methylimidazolium‐yl‐ethyl)‐3‐butylimidazolium dibromide (I***_***9***_ ***)***: ^1^H NMR (400 MHz, D_2_O, ppm), δ: 7.54 (m, 6H, Ar−H), 4.21 (t, ^3^ *J*_HH_=8 Hz, 6H, N−CH_2_), 3.91 (s, 3H, N−CH_3_), 1.83 (m, 2H, −CH_2_), 1.25 (m, 2H, −CH_2_), 0.92 (t, ^3^ *J*_HH_=8 Hz, 3H, −CH_3_). ^13^C NMR (100 MHz, D_2_O, ppm) δ: 125. 12 (N−CH−N), 122.78 (Ar−C), 49.35 (N−CH_3_), 36.60, 31.69, 19.42 (−CH_2_), 13.13 (−CH_3_).

#### 1,1’‐(Octane‐1,8‐diyl)‐bis(3‐methylimidazolium) bis(bromotrichloroferrate) (1)

Equimolar quantities of 1,1’‐(octane‐1,8‐diyl)‐bis(3‐methylimidazolium) dibromide (2 mmol) and iron(III) chloride (2 mmol) were used. Dark orange crystals; yield: 93 %; MP: 50 °C; anal. calcd (%) for [C_16_H_28_N_4_][FeCl_3_Br]_2_ (760.62): C, 25.26; H, 3.71; N, 7.37; found %: C, 25.71; H, 3.80; N, 7.28; FTIR (cm^−1^): 3145 (C−H_Aromatic_), 2958 (C−H_Aliphatic_), 1602 (C=N_Aromatic_), 1562 (C=C_Aromatic_), 1457 (C−N_Aromatic_), 1159 (C−N_Aliphatic_); *m*/*z* (ESI^+^): 138.11 (100 %) [C_4_(C_1_Im)]^+^, 193.17 (56 %) [C_7_(C_1_Im)]^+^, 275.22 (14 %) [C_8_(C_1_Im)_2_ ]^+^ (Table 1).

**Table 1:** Names and structures of DcILs.

Sample number	Name	Structure
1	1,1’‐(octcane‐1,8‐diyl)‐bis(3‐methylimidazolium) bis(bromotrichloroferrate)	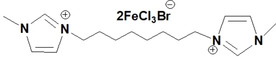
2	1,1’‐(pentane‐1,5‐diyl)‐bis(3‐methylimidazolium) bis(bromotrichloroferrate)	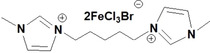
3	1,1’‐(ethane‐1,2‐diyl)‐bis(3‐methylimidazolium) bis(bromotrichloroferrate)	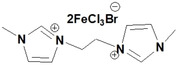
4	1,1’‐(octcane‐1,8‐diyl)‐bis(3‐butylimidazolium) bis(bromotrichloroferrate)	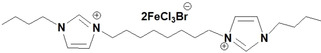
5	1,1’‐(pentane‐1,5‐diyl)‐bis(3‐butylimidazolium) bis(bromotrichloroferrate)	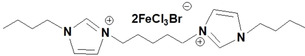
6	1,1’‐(ethane‐1,2‐diyl)‐bis(3‐butylimidazolium) bis(bromotrichloroferrate)	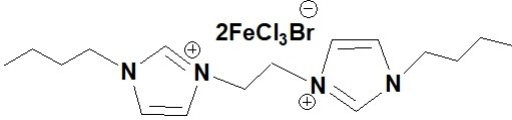
7	1‐(3‐methylimidazolium‐yl‐octyl)‐3‐butylimidazolium bis(bromotrichloroferrate)	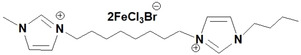
8	1‐(3‐methylimidazolium‐yl‐pentyl)‐3‐butylimidazolium bis(bromotrichloroferrate)	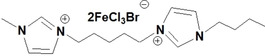
9	1‐(3‐methylimidazolium‐yl‐ethyl)‐3‐butylimidazolium bis(bromotrichloroferrate)	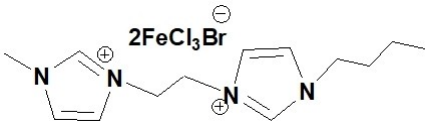

#### 1,1’‐(Pentane‐1,5‐diyl)‐bis(3‐methylimidazolium) bis(bromotrichloroferrate) (2)

Equimolar quantities of 1,1’‐(pentane‐1,5‐diyl)‐bis(3‐methylimidazolium) dibromide (2 mmol) and iron(III) chloride (2 mmol) were used. Dark orange crystals; yield: 91 %; MP: 56 °C; anal. calcd (%) for [C_13_H_21_N_4_][FeCl_3_Br]_2_ (717.70): C, 21.73; H, 3.09; N, 7.80; found %: C, 21.72; H, 3.21; N, 7.65; FTIR (cm^−1^): 3146 (C−H_Aromatic_), 2948 (C−H_Aliphatic_), 1610 (C=N_Aromatic_), 1563 (C=C_Aromatic_), 1455 (C−N_Aromatic_), 1162 (C−N_Aliphatic_); *m*/*z* (ESI^+^): 152.13(100 %) [C_5_(C_1_Im)]^+^, 233.17 (43 %) [C_2_(C_1_Im)_2_]^+^.

#### 1,1’‐(Ethane‐1,2‐diyl)‐bis(3‐methylimidazolium) bis(bromotrichloroferrate) (3)

Equimolar quantities of 1,1’‐(ethane‐1,2‐diyl)‐bis(3‐methylimidazolium) dibromide (2 mmol) and iron(III) chloride (2 mmol) were used. Dark orange crystals; yield: 94 %; MP: 145 °C; anal. calcd (%) for [C_10_H_16_N_4_][FeCl_3_Br]_2_ (676.48): C, 17.75; H, 2.38; N, 8.28; found %: C, 18.23; H, 2.61; N, 8.22; FTIR (cm^−1^): 3138 (C−H_Aromatic_), 2959 (C−H_Aliphatic_), 1597 (C=N_Aromatic_), 1541 (C=C_Aromatic_), 1454 (C−N_Aromatic_), 1163 (C−N_Aliphatic_); *m*/*z* (ESI^+^): 191.12 (100 %) [C_2_(C_1_Im)_2_]^+^, 273.05 (10 %) [C_2_(C_1_Im)(Im)]^+^.

#### 1,1’‐(Octane‐1,8‐diyl)‐bis(3‐butylimidazolium) bis(bromotrichloroferrate) (4)

Equimolar quantities of 1,1’‐(octane‐1,8‐diyl)‐bis(3‐butylimidazolium) dibromide (2 mmol) and iron(III) chloride (2 mmol) were used. Dark orange liquid; yield: 89 %; MP: <−10 °C; anal. calcd (%) for [C_20_H_40_N_4_][FeCl_3_Br]_2_ (844.74): C, 31.28; H, 4.77; N, 6.63; found %: C, 31.50; H, 5.11; N, 6.53; FTIR (cm^−1^): 3131 (C−H_Aromatic_), 2954 (C−H_Aliphatic_), 1605 (C=N_Aromatic_), 1560 (C=C_Aromatic_), 1461 (C−N_Aromatic_), 1158 (C−N_Aliphatic_); *m*/*z* (ESI^+^): 180.16 (100 %) [C_4_(C_4_Im)]^+^, 303.25 (12 %) [C_8_(C_4_Im)]^+^, 359.20 (33 %) [C_8_(C_4_Im)_2_]^+^.

#### 1,1’‐(Pentane‐1,5‐diyl)‐bis(3‐butylimidazolium) bis(bromotrichloroferrate) (5)

Equimolar quantities of 1,1’‐(pentane‐1,5‐diyl)‐bis(3‐butylimidazolium) dibromide (2 mmol) and iron(III) chloride (2 mmol) were used. Dark orange liquid; yield: 91 %; MP: −5 °C; anal. calcd (%) for [C_19_H_34_N_4_][FeCl_3_Br]_2_ (802.72): C, 28.43; H, 4.27; N, 6.98; found %: C, 28.61; H, 4.34; N, 6.74; FTIR (cm^−1^): 3144 (C−H_Aromatic_), 2960 (C−H_Aliphatic_), 1603 (C=N_Aromatic_), 1560 (C=C_Aromatic_), 1457 (C−N_Aromatic_), 1156 (C−N_Aliphatic_); *m*/*z* (ESI^+^): 159.10 (100 %) [C_3_(C_4_Im)]^+^, 261.12 (76 %) [C_5_(C_4_Im)(Im)]^+^, 318.28 (18 %) [C_5_(C_4_Im)_2_]^+^.

#### 1,1’‐(Ethane‐1,2‐diyl)‐bis(3‐butylimidazolium) bis (bromotrichloroferrate) (6)

Equimolar quantities of 1,1’‐(ethane‐1,2‐diyl)‐bis(3‐butylimidazolium) dibromide (2 mmol) and iron(III) chloride (2 mmol) were used. Dark orange crystals; yield: 87 %; MP: 52 °C; MW: [C_16_H_28_N_4_][FeCl_3_Br]_2_ 764.64; 7.37; found %: C, 24.84; H, 3.75; N, 6.94; FTIR (cm^−1^): 3142 (C−H_Aromatic_), 2961 (C−H_Aliphatic_), 1601 (C=N_Aromatic_), 1551 (C=C_Aromatic_), 1441 (C−N_Aromatic_), 1160 (C−N_Aliphatic_); *m*/*z*(ESI^+^): 138.18 (100 %) [C_1_(C_4_Im)_2_]^+^, 219.16 (38 %) [C_2_(C_4_Im)(Im)]^+^, 275.22 (3 %) [C_2_(C_4_Im)_2_]^+^.

#### 1‐(3‐Methylimidazolium‐yl‐octyl)‐3‐butylimidazolium bis(bromotrichloroferrate) (7)

Equimolar quantities of 1‐(3‐methylimidazolium‐yl‐octyl)‐3‐butylimidazolium dibromide (2 mmol) and iron(III) chloride (2 mmol) were used. Dark orange liquid; yield: 91 %; MP: <−10 °C; anal. calcd (%) for [C_19_H_34_N_4_][FeCl_3_Br]_2_ (802.71): C, 28,43; H, 4.27; N, 6.98; found %: C, 28.74; H, 4.23; N, 6.81; FTIR (cm^−1^): 3131 (C−H_Aromatic_), 2928 (C−H_Aliphatic_), 1611 (C=N_Aromatic_), 1560 (C=C_Aromatic_), 1460 (C−N_Aromatic_), 1160 (C−N_Aliphatic_); *m*/*z* (ESI^+^): 125.10 (100 %) [C_3_(C_1_Im)]^+^, 180.16 (77 %) [C_7_(C_1_Im)(Im)]^+^, 237.23 (18 %) [C_8_(C_4_Im)]^+^, 318.24 (30 %) [C_8_(C_4_Im)(C_1_Im)]^+^.

#### 1‐(3‐Methylimidazolium‐yl‐pentyl)‐3‐butylimidazolium bis(bromotrichloroferrate) (8)

Equimolar quantities of 1‐(3‐methylimidazolium‐yl‐pentyl)‐3‐butylimidazolium dibromide (2 mmol) and iron(III) chloride (2 mmol) were used. Dark orange liquid; yield: 88 %; MP: 15 °C; anal. calcd (%) for [C_16_H_28_N_4_][FeCl_3_Br]_2_ (760.64): C, 25.26; H, 3.71; N, 7.37; found %: C, 25.52; H, 3.81; N, 7.28; FTIR (cm^−1^): 3144 (C−H_Aromatic_), 2958 (C−H_Aliphatic_), 1619 (C=N_Aromatic_), 1561 (C=C_Aromatic_), 1456 (C−N_Aromatic_), 1158 (C−N_Aliphatic_); *m*/*z* (ESI^+^): 138.18 (100 %) [C_1_(C_4_Im)]^+^, 219.16 (57 %) [C_5_(C_1_Im)(Im)]^+^, 275.20 (40 %) [C_5_(C_1_Im)C_4_Im)]^+^.

#### 1‐(3‐Methylimidazolium‐yl‐ethyl)‐3‐butylimidazolium bis(bromotrichloroferrate) (9)

Equimolar quantities of 1‐(3‐methylimidazolium‐yl‐ethyl)‐3‐butylimidazolium dibromide (2 mmol) and iron(III) chloride (2 mmol) were used. Dark orange viscous liquid; yield: 90 %; MP: 60 °C; anal. calcd (%) for [C_13_H_22_N_4_][FeCl_3_Br]_2_ (718.56): C, 21.73; H, 3.09; N, 7.80; found %: C, 22.20; H, 3.18; N, 7.87; FTIR (cm^−1^): 3143 (C−H_Aromatic_), 2959 (C−H_Aliphatic_), 1599 (C=N_Aromatic_), 1524 (C=C_Aromatic_), 1455 (C−N_Aromatic_), 1163 (C−N_Aliphatic_); *m*/*z* (ESI^+^): 177.11 (100 %) [C_2_(C_1_Im)(Im)]^+^, 117.07 (90 %) [C_1_(C_3_Im)]^+^, 234.22 (40 %) [C_2_(C_1_Im)(C_4_Im)]^+^.

### X‐ray crystallography

A suitable crystal for **1** was selected and mounted on a Bruker APEX‐II CCD single crystal X‐ray diffractometer using the microfocus CuKα X‐ray beam (*λ*=1.54178 Å). The crystal was kept at 273(2) K during data collection. Single‐crystal X‐ray diffraction data for **1** was collected on Bruker D8 venture equipped with CCD detector diffractometer and graphite monochromator with CuKα radiation. The integration and reduction of collected data were obtained by using SAINT (Bruker 1998) program.^[42]^ The data were corrected to Lorentz and polarization effect. Structure solution and refinement were accomplished using the *Olex2* program suite (Form II).^[43]^ The crystal structure was solved and refined by using direct methods and Fourier transformation techniques and further refinement was accomplished by the *ShelXS‐97* programmes,^[44]^ with full‐matrix least‐square calculation on F^2^. All non‐hydrogen atoms were refined anisotropically, while hydrogen atoms associated with the carbon atoms were refined isotropically in geometrically constrained positions [*U_iso_* (H)=1.2*U_eq_* (C)].

### Thermal stability

Thermogravimetric analysis was performed on a Netzsch Jupiter TGA system by which data were collected in air at a heating rate 10 °C/min in the range of 20 to 800 °C.

### Magnetic Susceptibility

The Evans balance was used to measure the magnetic susceptibility of the synthesized iron‐containing DcILs. This instrument works by having two permanent magnets equipped back‐to‐back and endorsed by the suspension strip. An optical transducer detects a deflection when the sample tube is inserted in between the two magnets. One of these two magnets has a coil attached with it, through which a counteracted current is passed. The sample exerts some force on the magnet which is proportional to the applied current to the coil to counter act the force and therefore allows for a reading to be taken.

For the paramagnetic material, the gram magnetic susceptibility χ_g_ must be a positive number and is calculated from the following Equation (1) 1

(1) $$\chi {}_{g}=l\;\times \;C{}_{Bal}\;\left(R\;\cdot \;R{}_{0}\right)\;/\;\left(m\;\times 10{}^{9}\right)$$

where *l*=length of sample in tube (cm), *C*_*Bal*=_balance calibration constant, R=the measurement taken with sample, R_0_=the measurement taken without sample, m=sample mass in grams.

From the gram magnetic susceptibility, molar magnetic susceptibility χ_m_ and *μ*_eff_ are calculated by using the following relationships [(Eq. (2) and Eq. (3)]: 2, 3

(2) $$\chi {}_{m}=\chi {}_{g}\;\cdot \;\mathrm{Molecular}\;\mathrm{weight}$$

(3) $$\mu {}_{\mathrm{eff}}=2.828\;\left(\chi {}_{m}\;\cdot \;T\right){}^{1/2}]$$

respectively.^[45]^ However, a correction must be made as magnetic field will be weakly repelled from the pair of electrons in compounds.^[46]^

## Results and Discussion

**Scheme 1:**
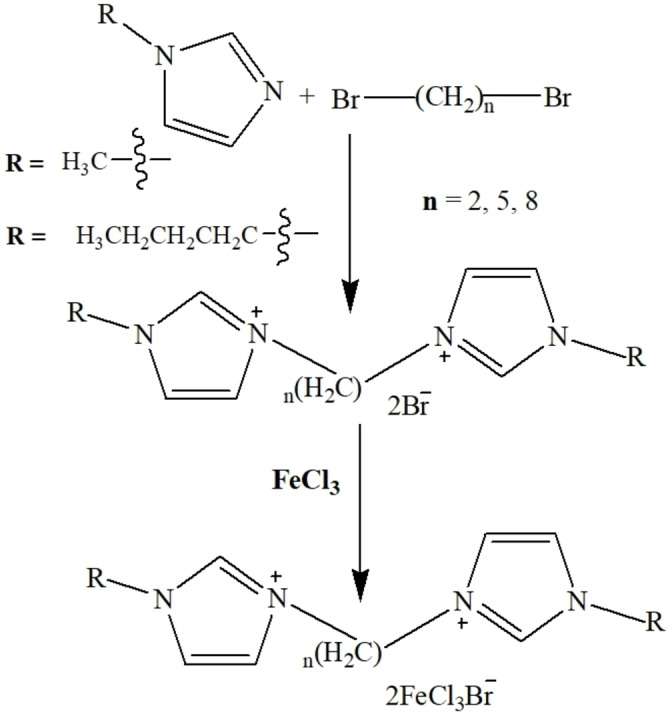
Synthetic pathway for dicationic ionic liquids **1**–**9**.

A series of compounds **1**–**9**, the symmetrical with methyl groups, symmetrical with butyl groups and asymmetrical with a linking alkyl chain varying from 2 to 5 to 8 carbon atoms long, have been synthesized as shown in Scheme 1. We split the sample set into three series. Compounds **1**–**3** have R1=R2=Me, and thus are symmetric, and the spacer length increases from compound **1** to compound **3**. Compounds **4** to **6** are also symmetric, but with R1=R2=Bu, and likewise the spacer length increases with increasing sample number. Lastly, compounds **7** to **9** are asymmetric, R1=Bu, R2=Me, with the spacer group increasing as with the previous series. The successful synthesis of DcILs having Br as counter anions [DcILs][Br]_2_ was confirmed using NMR spectroscopy, as shown in the Experimental Section and supplemented by electrospray analysis using mass spectrometry, operated in positive ionization mode with a HESI ion source. Their characteristic peaks are mentioned in the physicoanalytical data in the Experimental Section and spectra are shown in Figures S18–S26 in the Supporting Information which confirms the presence of the corresponding cations. The DcILs having [FeCl_3_Br]^−^ as counter anions were validated by electrospray mass analysis in negative ionization mode that demonstrated a base peak at 241.7 *m*/*z* for the [FeCl_3_Br]^−^ anion along with other isotopic peaks as shown in Figures S28–S29. The mass spectrum also contains peaks from [FeCl_4_]^−^ and [FeCl_2_Br_2_]^−^, indicating that halide exchange occurs, at least under mass spectrometry conditions, and likely in the sample as well. The corresponding analytical data of these synthesized DcILs are also well in accordance with the previous studies.^[41]^ UV‐Vis reflectance spectra (Figure S30) also demonstrated the characteristic absorption band edges around 620 nm for [FeCl_3_Br]^−^ and these results correspond very well with the literature.^[47]^

**Figure 1:**
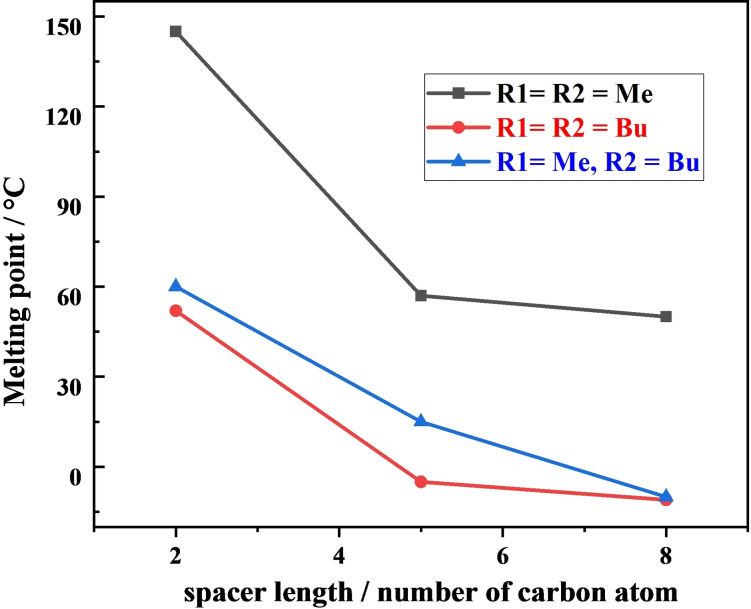
Variation in melting point with changing spacer length.

The strength of the ionic interaction between the cation and anion influences the melting point of ionic liquids.^[48]^ The lattice energy depends on the molecular symmetry, intermolecular forces of attraction, and conformational degrees of freedom.[^49^, ^50^] Figure 1 shows the melting points that we have recorded for compounds **1** to **9**. Within each series, the melting point decreases with increasing spacer length. Similarly, when the substituent chain length at the 3 position of the imidazole ring increases, this results in added conformation degree of freedom and melting point of the DcILs decreases.

However, in case of the asymmetric DcILs series, (compounds **7** to **9**), which have different substituents at the 3 position of the imidazolium ring on both sides, the melting point is closer to the compounds having R1=R2=Bu, being considerably lower than those having R1=R2=Me for equivalent spacer chain lengths. If the melting point depended on the size of the cations alone (e. g. by considering the number of carbon atoms in each cation), the asymmetric series melting points might be expected to fall midway between the melting points of the two symmetric analogues with the same chain lengths. In fact, the asymmetric compounds show melting points much lower than this, very close to the melting points of the larger R1=R2=Bu series. This shows the influence of the asymmetry in the cation in depressing the melting point of compounds **7** to **9**. While we keep the anion constant in this work, changing the anion is also expected to have an effect on the melting point. The higher polarizability of [FeBr_4_]^−^ would result in the stronger dispersion forces as compared to [FeCl_4_]^−^ leading to an elevation in melting points.^[51]^ So, it could be concluded from this detail that sequence of melting point in anions varies in the manner of [FeBr_4_]^−^>[FeCl_3_Br]^−^>[FeCl_4_]^−^ which is well in accordance with the previous reports where glyme‐lithium complex cation has [FeBr_4_]^−^, [FeCl_3_Br]^−^ and [FeCl_4_]^−^ as counter anions and their melting points were ∼80, 45 and 28 °C, respectively.[^52^, ^53^]

Elemental analysis infers that the calculated and observed values for C, H and N are well in agreement to each other as given in the physicoanalytical data in the Experimental Section. The calculated and observed values of C, H and N deviate within the range of ±0.50 %.

**Figure 2:**
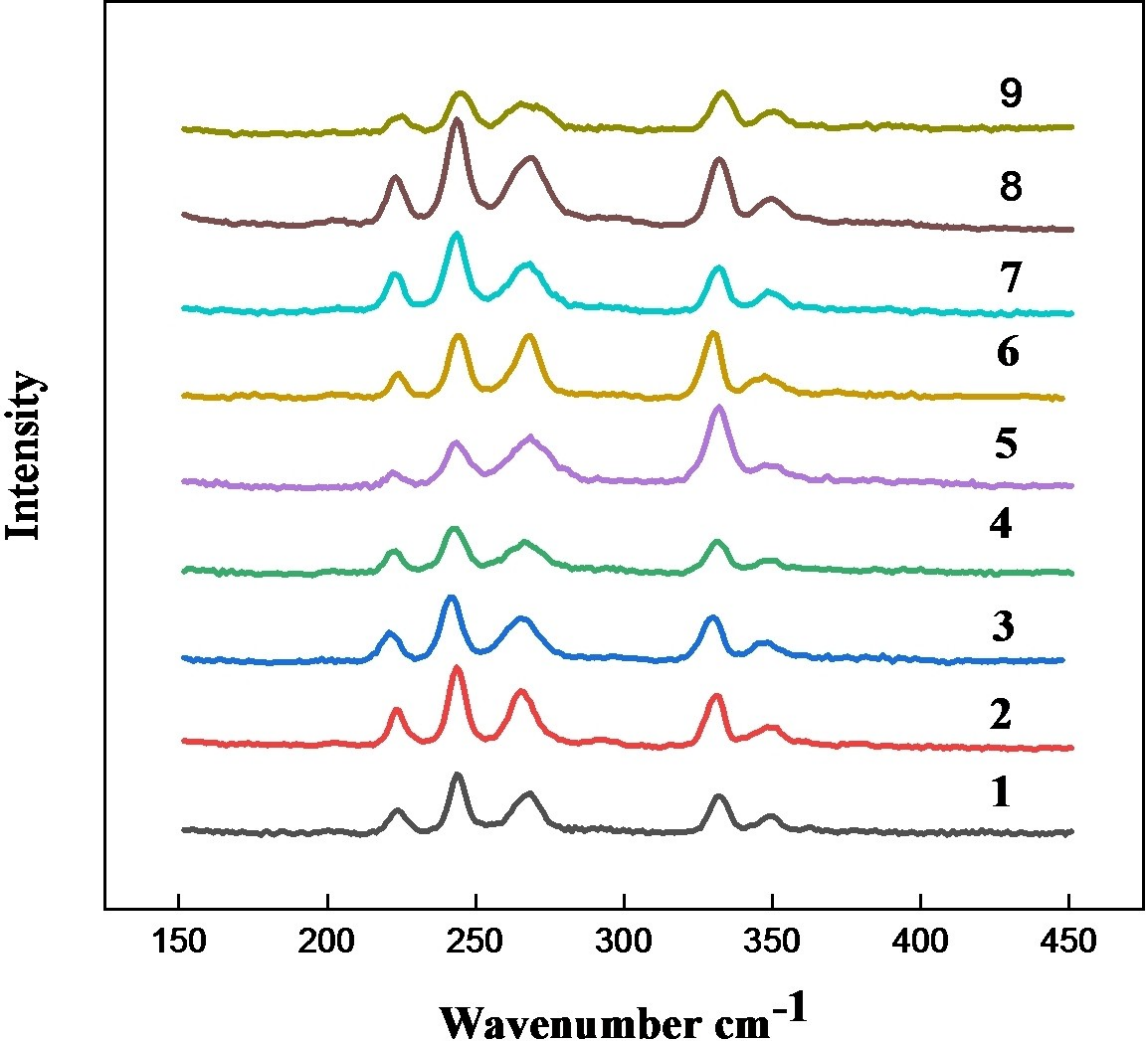
Raman spectra for compounds **1**–**9**.

The Raman spectra of all compounds (Figure 2) are dominated by vibrations of the asymmetric [FeCl_3_Br]^−^ anion. The spectra are almost identical in the 150–450 cm^−1^ region. The [FeCl_3_Br]^−^ anion exhibits *C*_3*v*_ symmetry.^[54]^ According to group theory and point group analysis, two Raman‐active Fe−Cl stretches, and one Raman‐active Fe−Br stretch are predicted.^[55]^ However, in practice, two bands are observed at 332 cm^−1^ and 350 cm^−1^, which due to their higher frequency are assigned to the Fe−Cl stretches, and a group of lower frequency bands at 223, 244 and 268 cm^−1^, are likewise assigned to Fe−Br stretching vibration^[56]^ as shown in Figure 2. The fact that more vibrational bands are observed than predicted for the *C*_3*v*_ anion suggests that, in fact, a mixture of different anions may be present, with a range of halide compositions, giving an overall average composition of [FeCl_3_Br]. This is likely due to dynamic halide exchange between anions. This is supported by single‐crystal XRD data discussed in the following.

### Crystal structure determination

The single‐crystal X‐ray analysis for **1** was carried out to substantiate the structure of the dicationic IL and the data illustrates that it crystalizes in the monoclinic crystal system with the *P*2_1_/*c* space group. The ORTEP diagram (Figure 2) depicts that the unit cell consists of cationic and anionic part and that the cation is composed of a dimethylimidazolium core linked to a chain of eight carbon atoms (octyl). The anion consists of bromotrichloroferrate(III) assuming a distorted tetrahedral geometry for the species. The crystallographic and refinement parameters for **1** are given in Table 2 and their selected bond lengths and angles are listed in Table 3.

**Table 2:** Crystallographic data and structure refinement details for **1**.

Identification code	**1**
Empirical formula	C_16_H_28_Br_2_Cl_6_Fe_2_N_4_
Formula weight	760.64
Temperature/K	273(2)
Crystal system	monoclinic
Space group	*P*2_1_/*c*
a/Å	7.6279(2)
b/Å	13.2848(3)
c/Å	14.7813(4)
α/°	90
β/°	94.0220(10)
γ/°	90
Volume Å^−3^	1494.18(7)
Z	2
ρ_calc_g/cm^3^	1.691
μ/mm^−1^	15.954
F(000)	752.0
Crystal size/mm^3^	0.45×0.28×0.19
Radiation	CuKα (*λ*=1.54178)
2Θ range for data collection/°	11.63 to 136.484
Index ranges	−9≤h≤8, −16≤k≤15, −17≤l≤16
Reflections collected	17548
Independent reflections	2731 [R_int_=0.0644, R_sigma_=0.0408]
Data/restraints/parameters	2731/0/174
Goodness‐of‐fit on F^2^	1.103
Final R indexes [I>=2σ (I)]	R_1_=0.0590, wR_2_=0.1473
Final R indexes [all data]	R_1_=0.0624, wR_2_=0.1520
Largest diff. peak/hole/e Å^−3^	0.90/−0.90

**Table 3:** Selected bond lengths (Å) and bonds angles (°) for **1**

Bond lengths/Å	
Fe1−Cl1	2.257(6)
Fe1−Cl2	2.226(8)
Fe1−Cl3	2.301(8)
Fe1−Cl4	2.275(5)
Fe1−Br1	2.251(2)
Fe1−Br2	2.309(9)
Fe1−Br3	2.175(8)
Fe1−Br4	2.036(19)

Bond Angles/°	
Cl1−Fe1−Cl3	110.0(2)
Cl1−Fe1−Cl4	107.2(2)
Cl2−Fe1−Cl1	105.6(3)
Cl2−Fe1−Cl3	119.5(3)
Cl2−Fe1−Cl4	107.2(3)
Cl4−Fe1−Cl3	106.7(2)
Br1−Fe1−Br2	109.4(2)
Br3−Fe1−Br1	112.1(2)
Br3−Fe1−Br2	102.5(3)
Br4−Fe1−Br1	114.3(6)
Br4−Fe1−Br2	108.2(7)
Br4−Fe1−Br3	109.7(7)
C5−N1−C4	125.7(4)
C6−N1−C4	126.6(4)

The two aromatic methylimidazole rings of the cation are in twisted configuration around the chain linkage forming the angles (C_4_⋅⋅⋅N_1_⋅⋅⋅C_5_) 125.7(4)° and (C_4_⋅⋅⋅N_1_⋅⋅⋅C_6_) 126.6(4)°. The central iron atom is coordinated with chloride and bromide in a 3 : 1 ratio and they can co‐exist at each halide site with varying occupying probability at different position in tetrahedral geometry as listed in Table 4. This supports the conclusions from the Raman spectroscopy that a mixture of different anions is present. The bond lengths for Fe−Cl and Fe−Br are in the range of 2.226(8)–2.301(8) Å and 2.036(19)–2.309(9) Å, respectively, and are comparable to values found in the literature.^[57]^ The mean value for bond angles of Cl−Fe−Cl and Br−Fe−Br comes out to be same 109.3(3)° which is good in agreement with the mentioned geometry of the species and tallies with reported values in the previous studies.^[35]^

**Table 4:** Atomic Occupancy for **1**.

Atom	*Occupancy*	Atom	*Occupancy*	Atom	*Occupancy*
Cl1	0.7	Cl2	0.7	Cl3	0.7
Cl4	0.9	Br1	0.3	Br2	0.3
Br3	0.3	Br4	0.1		

**Figure 3:**
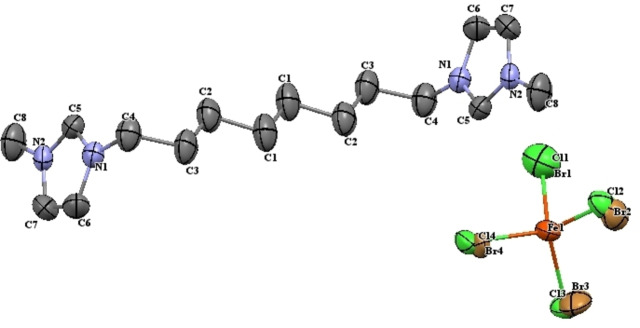
ORTEP diagram of the asymmetric unit for **1**.

**Figure 4:**
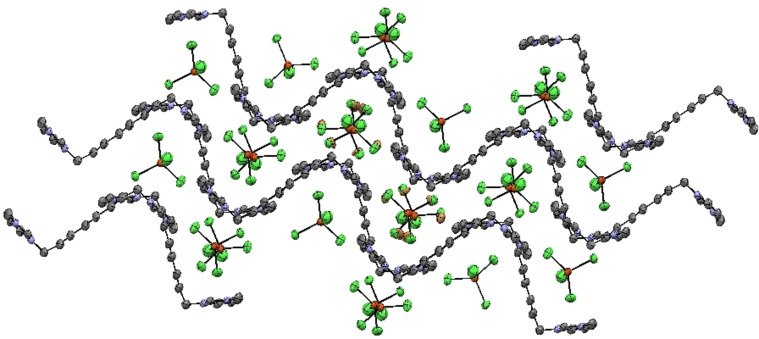
Packing diagram for **1** viewed along the crystallographic axis *a*.

The cations and anions interact with each other through C−H⋅⋅⋅Cl/Br, π(C−C)⋅⋅⋅Cl/Br and π(C−N)⋅⋅⋅Cl/Br contacts. The cations are arranged in zigzag fashion along the crystallographic *a*‐axis and in between these layers, anions are present which interact with cations to give a 3D packing network as depicted in Figure 4. The cation and anions also have hydrogen bonding in the form of C−H⋅⋅⋅Cl/Br interactions. The ORTEP (Figure 3) and packing diagram (Figure 4) are drawn at the 50 % probability level and hydrogen atoms are omitted to improve the clarity of the diagrams.

### Supramolecular features

**Figure 5:**
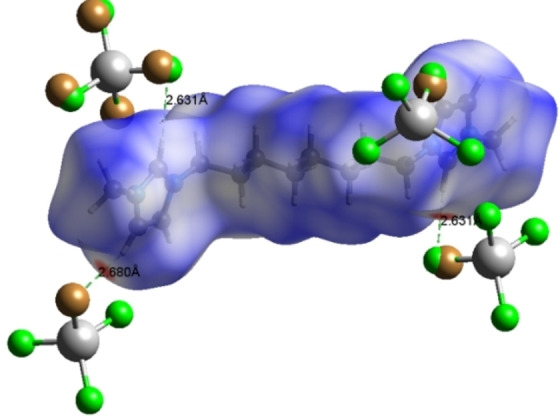
The 2D Hirshfeld surface generated over the region of cationic part and packing mode of cationic part with the anion.

**Figure 6:**
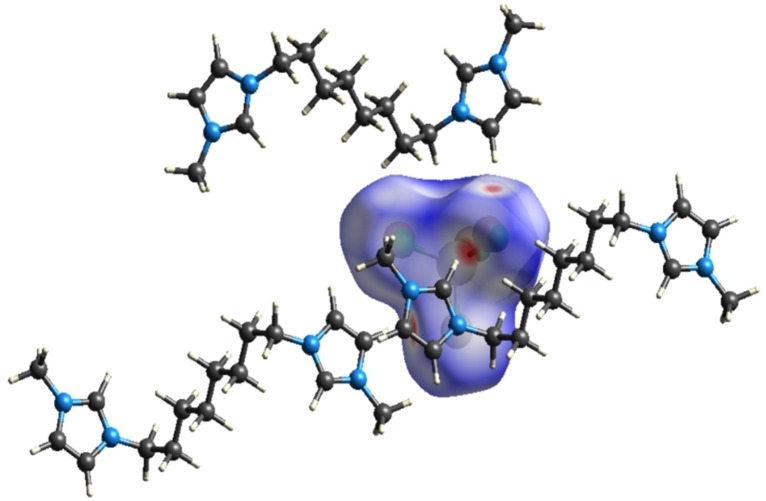
The 2D Hirshfeld surface of **1** generated over the anionic region and packing mode of anionic part with ligand.

PLATON analysis revealed the presence of non‐classical hydrogen bonds (Table 5), which are involved in the unit cell packing of **1**.^[58]^ The intermolecular interactions C(5)−H(5)⋅⋅⋅Cl(1), and C(7)−H(7)⋅⋅⋅Cl(2) intermolecular interactions were responsible for unit cell packing having the contact distance of 2.75 and 2.80 Å. These H−Cl‐based interactions are responsible for chain elongation via *c*‐axis.^[59]^ Besides this comparatively weak interaction, Fe(1)−Cl(2)⋅⋅⋅Cg(1) has also been found to be present in PLATON analysis having a distance of 3.6308 Å. The Hirshfeld surface analysis is a valuable tool for analysis of non‐covalent interaction both qualitatively and quantitatively.^[60]^ It has been utilized for detailed analysis of various types of interactions. The 2D Hirshfeld surface was generated using normal parameters and shows bright red spots at the regions of H−Cl, and H−Br contact which indicates the involvement of H−Cl, and H−Br contact in unit cell packing (Figure 5). Further analysis reveals the involvement of [FeCl_3_Br]^−^ in making interaction with other three neighbouring cationic moieties (Figure 6).

**Table 5:** Hydrogen bonds for **1**.

D	H	A	d(D−H)/Å	d(H−A)/Å	d(D−A)/Å	D−H−A/°
C3	H3AB	Br3^1^	0.97	3.05	4.017(11)	175.0
C4	H4A	Br4	0.97	3.22	4.08(2)	148.7
C4	H4AB	Br2^2^	0.97	3.07	3.821(11)	135.6
C5	H5	Cl4	0.93	2.76	3.560(6)	145.3
C5	H5	Br4	0.93	2.80	3.60(2)	145.5
C6	H6	Cl4^2^	0.93	2.90	3.717(7)	146.8
C6	H6	Br4^2^	0.93	2.88	3.65(2)	141.3
C7	H7	Cl1^3^	0.93	2.81	3.698(8)	159.2
C8	H8 C	Br2^4^	0.96	3.05	3.991(12)	168.2

**Figure 7:**
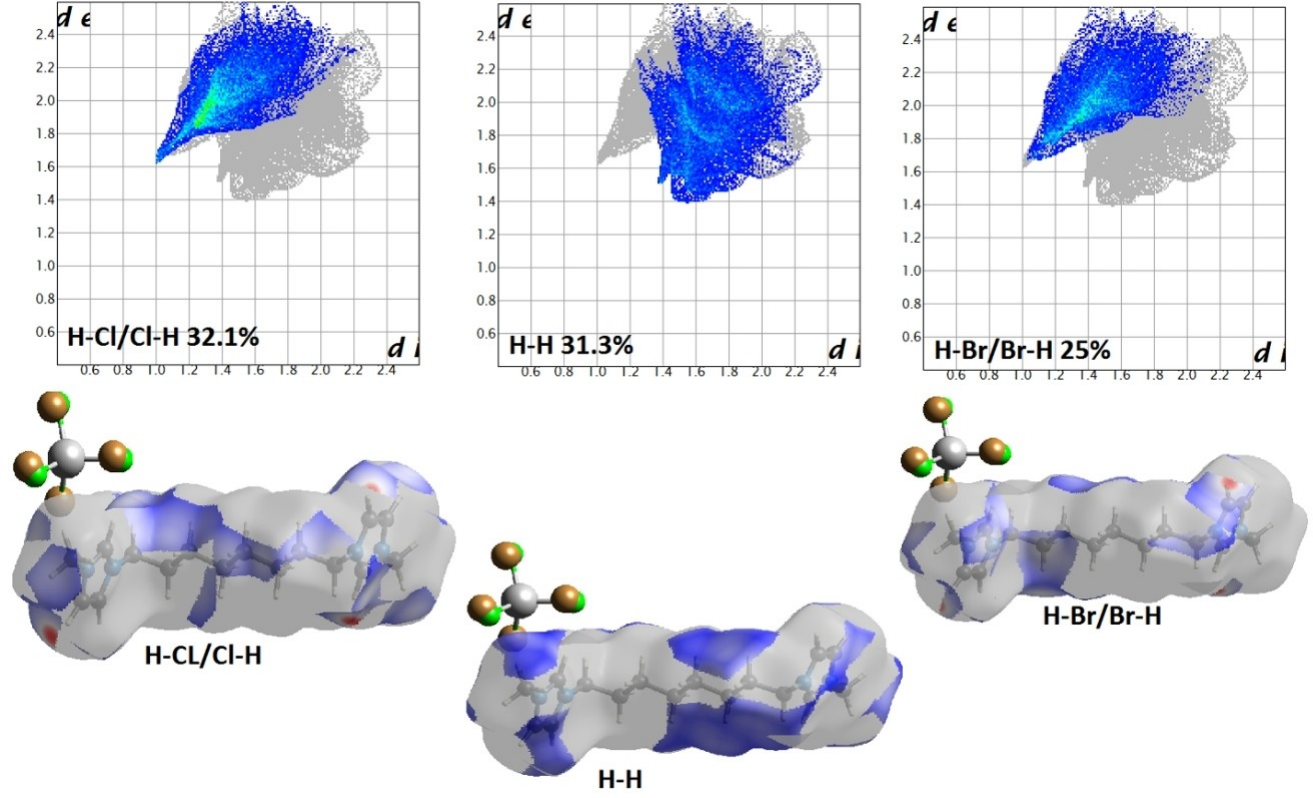
The 2D Hirshfeld fingerprint plot of **1**.

The 2D fingerprint plots analysis (Figure 7) revealed the interaction in a sense of quantitative analysis, and the maximum contribution was found to be of H−Cl/Cl−H character which is 32.1 % in overall unit cell packing. Moreover, H−H, and H−Br/Br−H were found to have 31.3 and 25 % contribution towards unit cell packing, respectively.

### Solubility of dicationic ILs in different solvents

The solubility data for the synthesized DcILs in various solvents are summarized in Table 6. All the ILs exhibited good solubility in methanol, ethanol, acetonitrile and DMSO which was due to favourable interactions between these ILs and polar solvents.^[61]^ They are insoluble in non‐polar organic solvents including diethyl ether, chloroform, and n‐hexane. The dibromide containing dicationic ILs (**I_1_**–**I_9_**) (i. e., the intermediate compounds shown in Scheme 1), when isolated, are partially soluble in acetone and dichloromethane, however, by incorporating replacing the Br^−^ anion with [FeCl_3_Br]^−^, that is, by formation of compounds **1**–**9**, they became completely soluble in these solvents. Attractively, DcILs could form a homogenous system with relatively low amount of water and acetonitrile at room temperatures. It has been observed that their miscibility decreased as the chain linkage of dicationic ILs increased. It may be due to the higher hydrophobic character of chain linkage or lower symmetry of cation that decreases the miscibility.

**Table 6:** Solubility of dicationic ILs in water and common organic solvents.^[a]^

DcILs	H_2_O	MeOH	EtOH	ACN	DMSO	Acetone	DCM	THF	EtOAc	Et_2_O	n‐Hex
I_1_	++	++	++	++	++	±	±	− −	− −	− −	− −
I_2_	++	++	++	++	++	±	±	− −	− −	− −	− −
I_3_	++	++	++	++	++	±	±	− −	− −	− −	− −
I_4_	++	++	++	++	++	±	±	− −	− −	− −	− −
I_5_	++	++	++	++	++	±	±	− −	− −	− −	− −
I_6_	++	++	++	++	++	±	±	− −	− −	− −	− −
I_7_	++	++	++	++	++	±	±	− −	− −	− −	− −
I_8_	++	++	++	++	++	±	±	− −	− −	− −	− −
I_9_	++	++	++	++	++	±	±	− −	− −	− −	− −
1	++	++	++	++	++	++	++	±	±	− −	− −
2	++	++	++	++	++	++	++	±	±	− −	− −
3	++	++	++	++	++	++	++	±	±	− −	− −
4	++	++	++	++	++	++	++	±	±	− −	− −
5	++	++	++	++	++	++	++	±	±	− −	− −
6	++	++	++	++	++	++	++	±	±	− −	− −
7	++	++	++	++	++	++	++	±	±	− −	− −
8	++	++	++	++	++	++	++	±	±	− −	− −
9	++	++	++	++	++	++	++	±	±	− −	− −

[a] **++**: miscible, **±**: slightly miscible, − −: immiscible

### Thermal analysis

**Figure 8:**
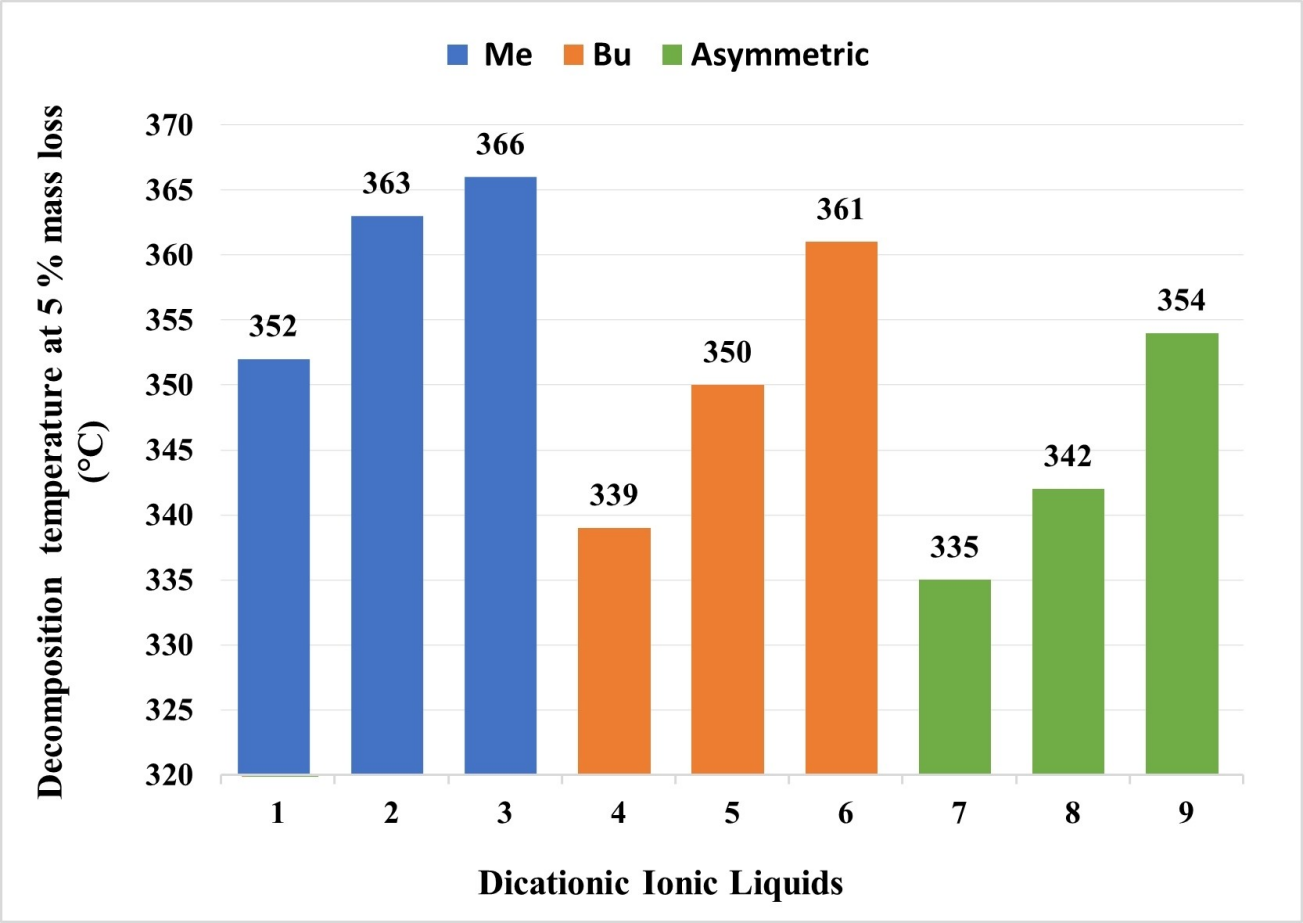
Ramped temperature TGA (10 °C min^−1^, Al_2_O_3_ pans) of DcILs **1**–**9** at 5 % mass loss.

The ILs are generally purported as thermally stable due to their higher decomposition temperature as compared with many organic solvents.^[62]^ The thermal stability of DcILs was assessed by using thermogravimetric analysis (TGA) where mass loss was monitored by continuously increasing the temperature (Figure 8, Table 7). Comparing the results, it can be inferred from the data that the thermal stability of **1** to **3**, **4** to **6** and **7** to **9** increases with the decrease in the alkyl chain length between the two cations. This is possibly because of increase in stability of free radicals and linear non aromatic carbocation with increase in their chain length that have a tendency to be a better leaving group in course of heating and hence, advocating the breaking of the C−N bond.^[63]^ Apparently, no exceptional variance among the thermal stabilities of symmetric and asymmetric DcILs have been determined which indicates that the nature of cations on both side of the alkyl chain does not significantly affect the thermal stability of DcILs but rather the alkyl chain length between the two cations.

**Table 7:** Thermal stability of DcILs.

DcILs	Thermal stability [°C]			
	99 % mass	95 % mass	90 % mass	85 % mass
1	325	352	360	366
2	327	363	380	388
3	331	366	382	391
4	312	339	353	367
5	319	350	370	379
6	336	361	379	395
7	321	335	342	371
8	333	342	343	352
9	377	352	358	369

### Magnetism

Both symmetric and asymmetric iron‐containing ILs exhibited paramagnetic behaviour at 294 K and follow the Curie‐Weiss law with the Weiss temperature being higher than 20 K (Table 8). The effective paramagnetic moment value as mentioned in the literature is [*μ*_eff_]=5.9 *μ*_B_ for a high‐spin d^5^ (S=5/2), Fe^3+^ system.^[64]^ The products of molar susceptibility and temperature (χ_mol_⋅T) for these synthesized ILs were calculated and found in between 4.05 to 4.79 emu mol^−1^ K Oe^−1^ (NB this value is quoted per mol of magnetic Fe centre, not per mole of DcIL formula unit) which is in accord with the reported value (4.37 emu mol^−1^ K Oe^−1^) for the Fe^3+^ system.^[65]^

**Table 8:** Gram and molar susceptibilities along with effective magnetic moments for **1**–**9** at 294 K.

Sample number	χ_g_ [emu g^−1^ Oe^−1^]	χ_mol_^(corr)^ [emu mol^−1^ Oe^−1^]	χ_mol_ T [emu mol^−1^ K Oe^−1^)	*μ*_eff_/*μ*_B_
1	39×10^−6^	0.015	4.48	4.22
2	45×10^−6^	0.016	4.785	4.36
3	41×10^−6^	0.014	4.085	4.03
4	35×10^−6^	0.0145	4.045	4.405
5	39×10^−6^	0.0155	4.675	4.315
6	36×10^−6^	0.014	4.085	4.03
7	36×10^−6^	0.0145	4.325	4.14
8	38×10^−6^	0.0145	4.33	4.15
9	42×10^−6^	0.015	4.47	4.165

It is possible to exploit the physico‐chemical properties of DcILs like hydrophobicity, solubility and thermal stability, which are significant for their applications in various fields at the same time maintaining their para‐magneticity.^[66]^

## Conclusions

A series of symmetric and asymmetric dicationic ionic liquids containing [FeCl_3_Br]^−^ as anions, and cations based on methyl imidazole and butyl imidazole linked through varying alkyl chains, have been successfully synthesized. Single‐crystal XRD analysis and other spectroscopic techniques were used to fully characterize the synthesized compounds, along with determination of their physico‐chemical and magnetic properties. The DcILs could be of importance in the design of heterogenous and homogenous systems with water and other organic solvents which may benefit applications like efficient isolation and separation of various products. They displayed very good thermal stability over a wide range of temperature which may give them a new prospect in numerous practical applications like heat transfer fluids, extracting agents, lubricants, electrolytes, and high temperature solvents, among others. These magnetic DcILs may have potential applications in the generation of membranes for selective and tuneable transport of gases on applying of external magnetic fields and magneto‐structural correlations close to the room temperature will be of great interest in wide community of scientific research in near future. This approach to design DcILs has also offered general templates that may possibly be used within a broader range of molecular architectures to tune their properties for targeted applications

## Supplementary Data

Deposition Number 2150332 (for **1**) contains the supplementary crystallographic data for this paper. These data are provided free of charge by the joint Cambridge Crystallographic Data Centre and Fachinformationszentrum Karlsruhe Access Structures service.

## Conflict of interest

The authors declare no conflict of interest.

1

## Supporting information

As a service to our authors and readers, this journal provides supporting information supplied by the authors. Such materials are peer reviewed and may be re‐organized for online delivery, but are not copy‐edited or typeset. Technical support issues arising from supporting information (other than missing files) should be addressed to the authors.

Supporting Information

## Data Availability

The data that support the findings of this study are available from the corresponding author upon reasonable request.
