# Supplementary material for: Physico‐Chemical Properties of Magnetic Dicationic Ionic Liquids with Tetrahaloferrate Anions
Source: ChemistryOpen. 2023 Jan 4;12(1):e202200229. doi: 10.1002/open.202200229 (PMC9812754; doi:10.1002/open.202200229)
Supplement: Supplementary file 1 — Supporting Information [file OPEN-12-e202200229-s001.pdf]

ChemistryOpen

Supporting Information

Physico-Chemical Properties of Magnetic Dicationic Ionic Liquids with Tetrahaloferrate Anions

Anham Zafar, Imtiaz-ud-Din, Robert G. Palgrave,* Haji Muhammad, Sammer Yousuf, and Tim Evans

Contents

1. NMR Spectrocpoy 2
2. Mass spectrometry ESI-MS (positive ion mode(+ve)........ 12
3. X-ray Crystallography 17
4. TGA Analysis........ 22
5. Mass spectrometry ESI-MS (Negative ion mode(-ve)........ 23
6. UV-vis spectrophotometry 24
7. Magnetic Properties........ 24

1. NMR Spectrocpoy

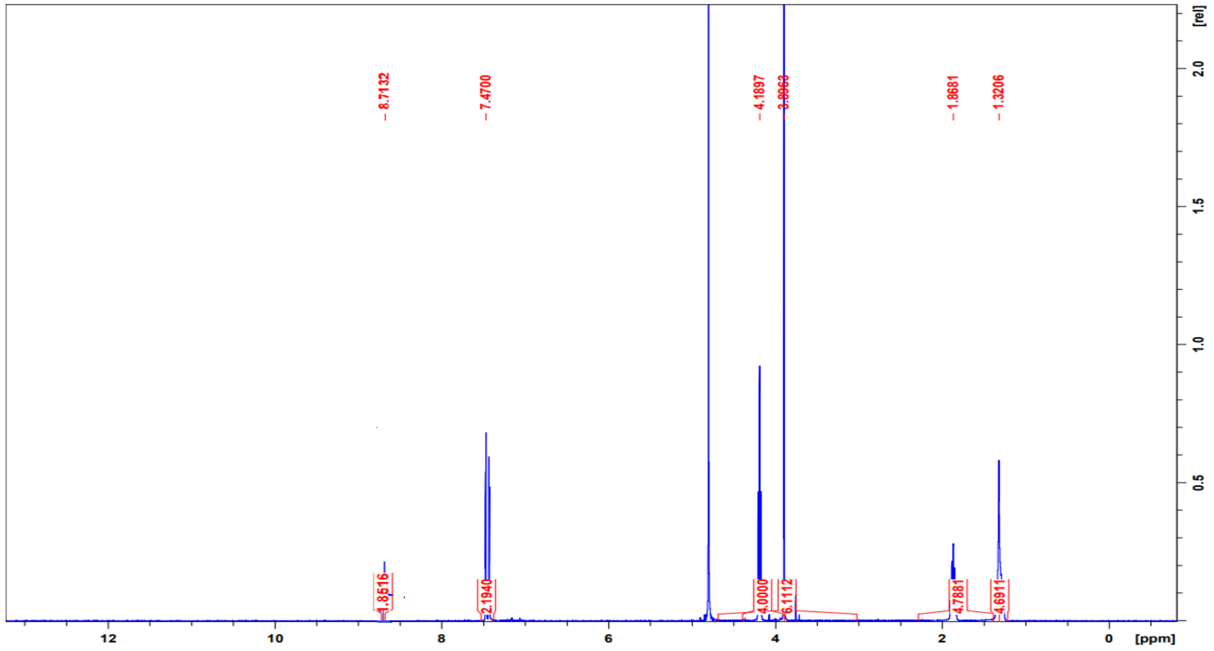

Fig.S1. ^{1}H NMR spectrum for (**1**)

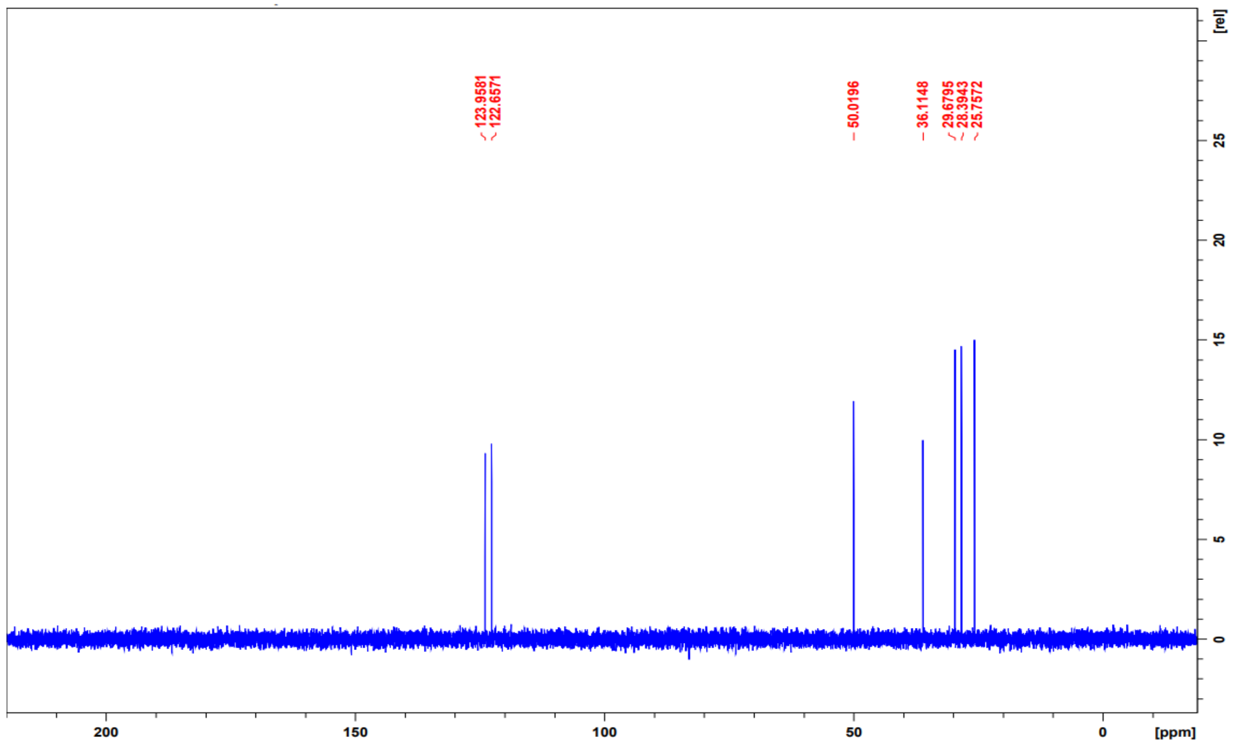

Fig.S2. ^{13}C NMR spectrum for (**1**)

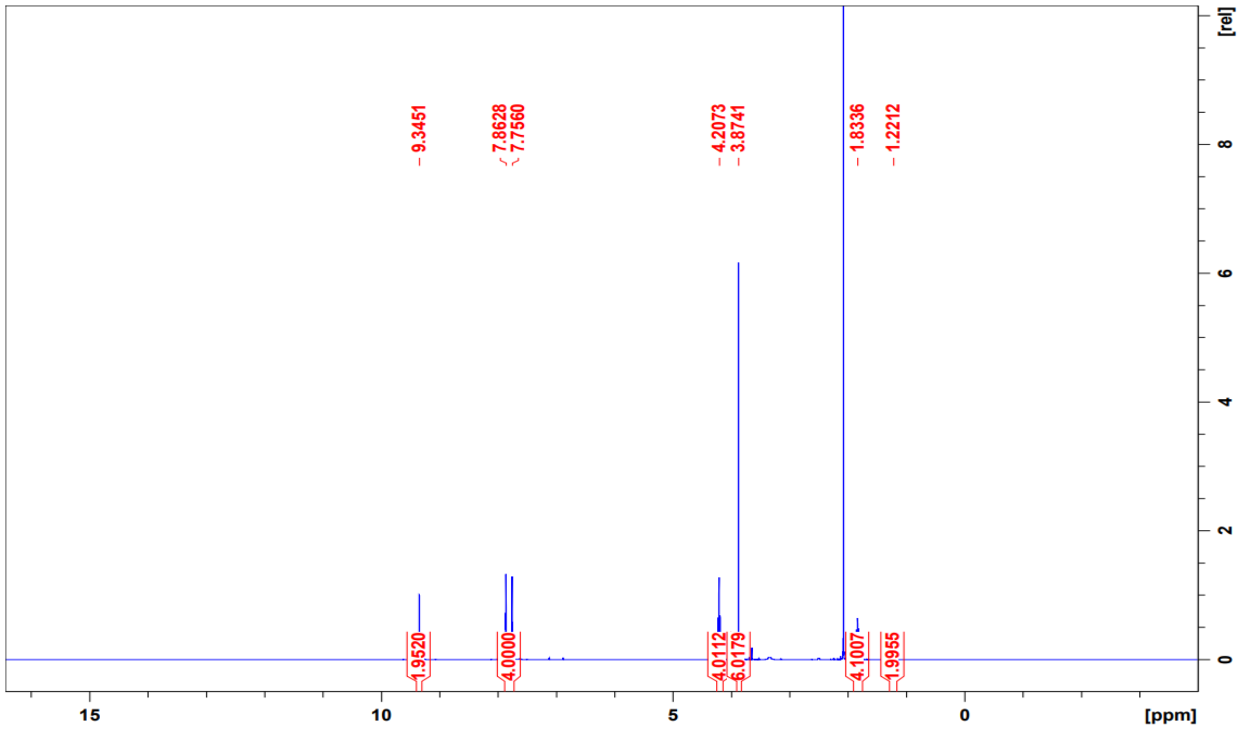

Fig.S3. ^{1}H NMR spectrum for (**2**)

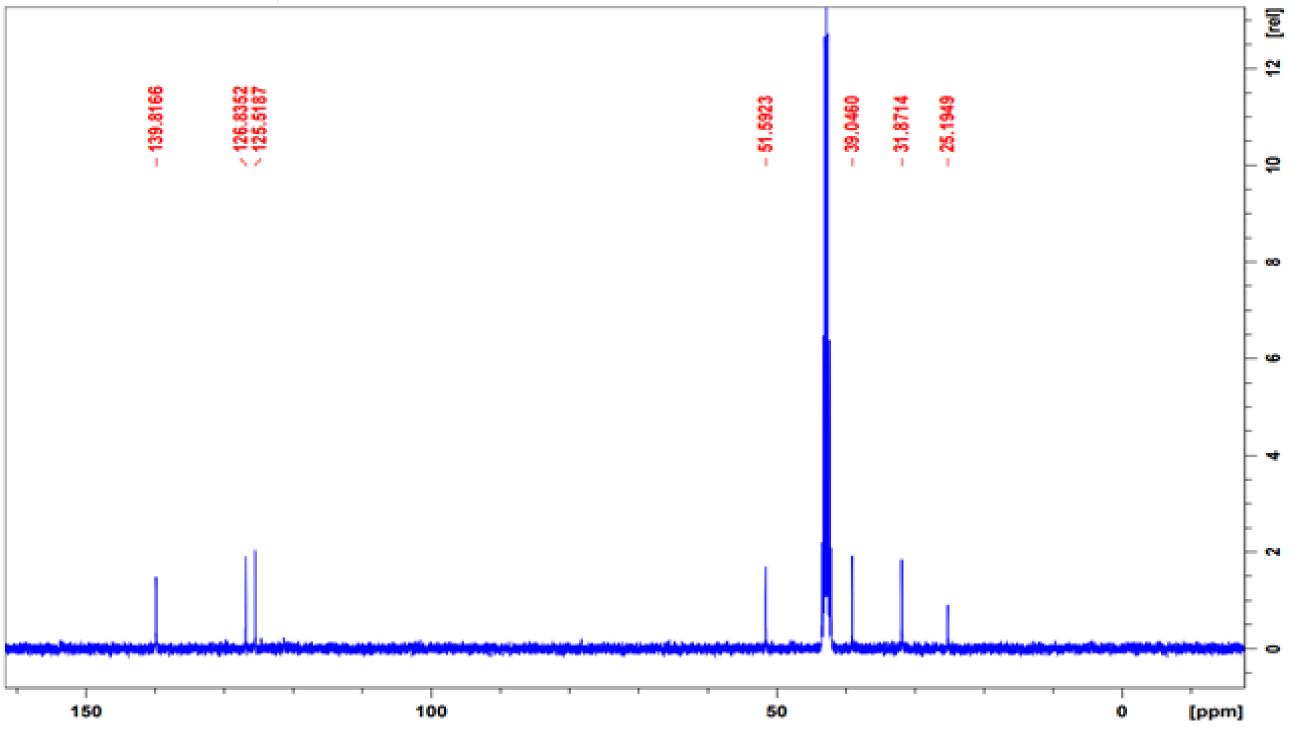

Fig.S4. ^{13}C NMR spectrum for (**2**)

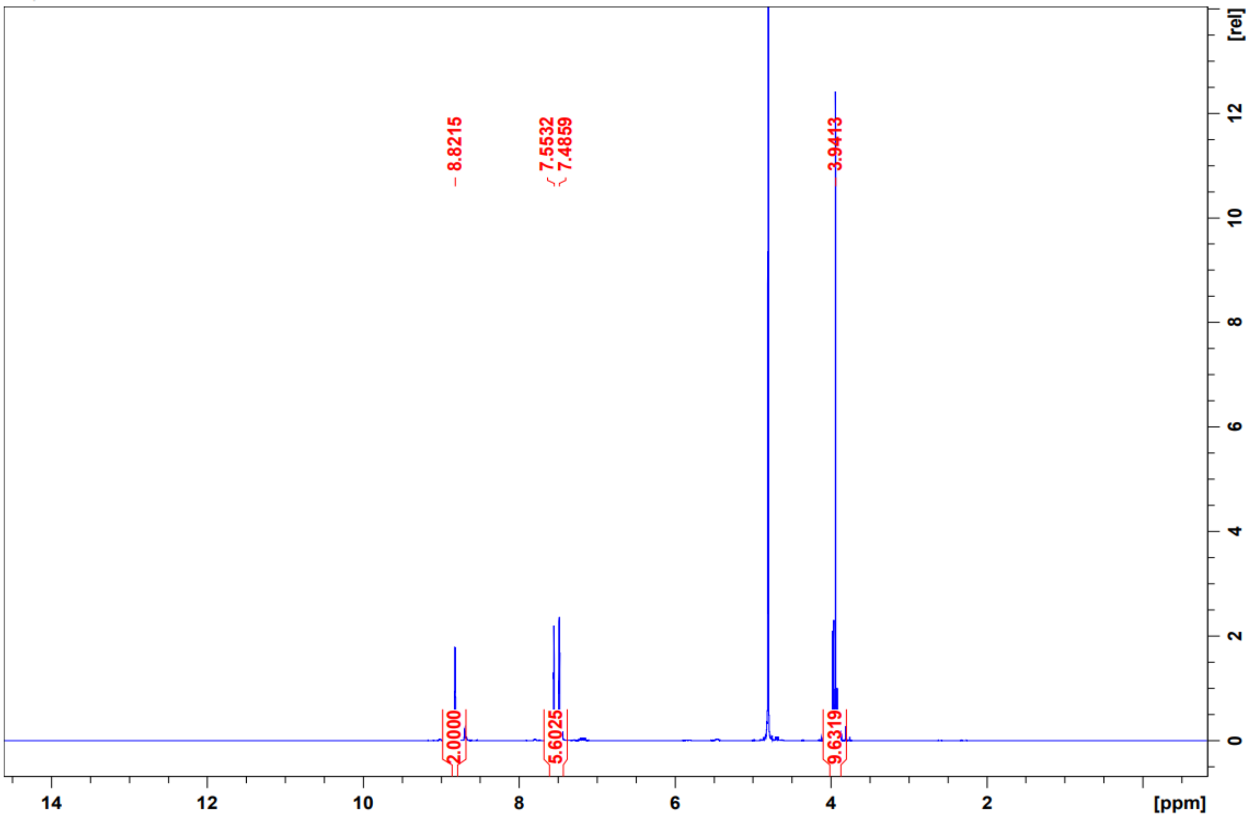

Fig.S5. ^{1}H NMR spectrum for (**3**)

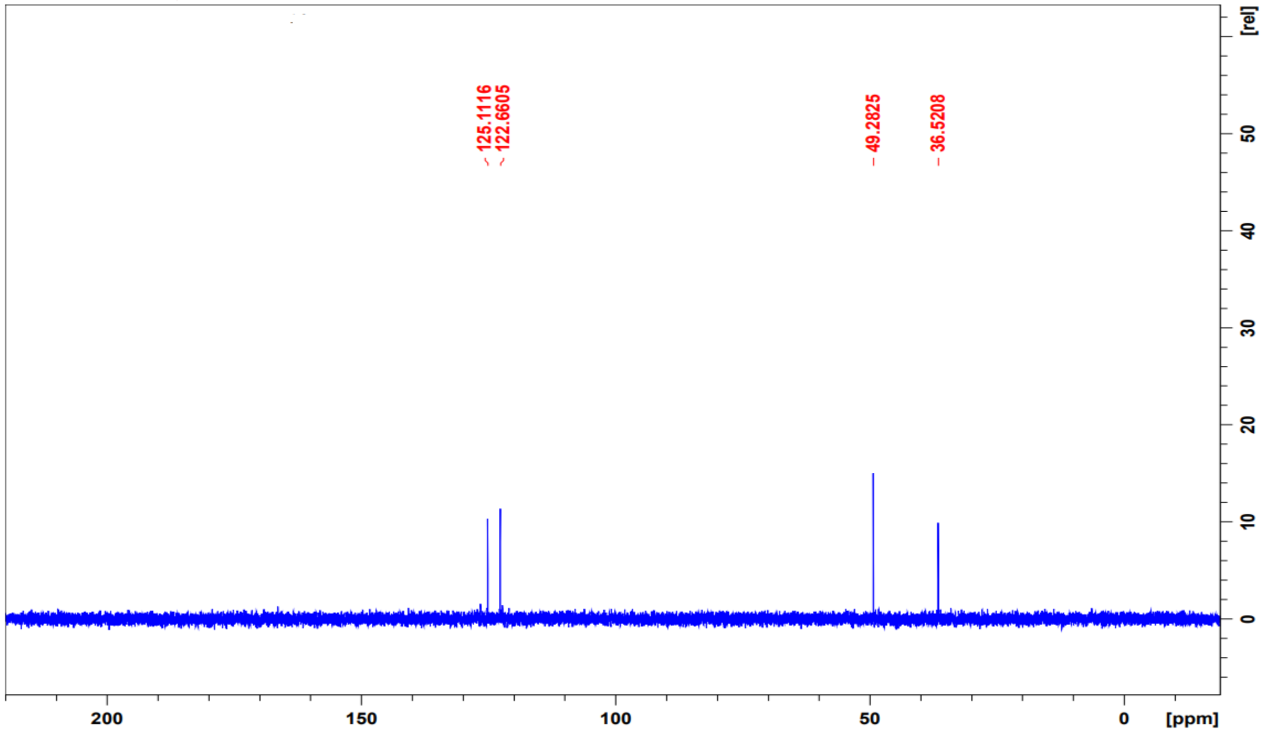

Fig.S6. ^{13}C NMR spectrum for (**3**)

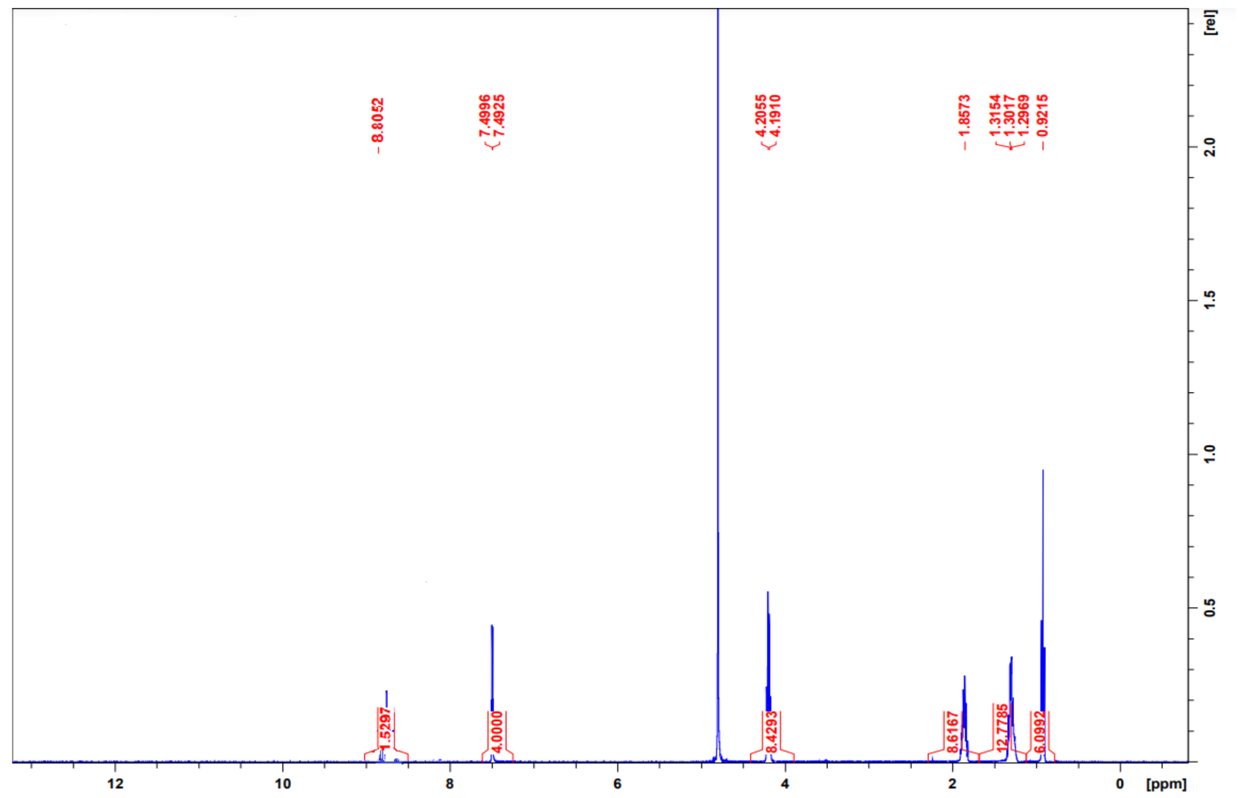

Fig.S7. ^{1}H NMR spectrum for (**4**)

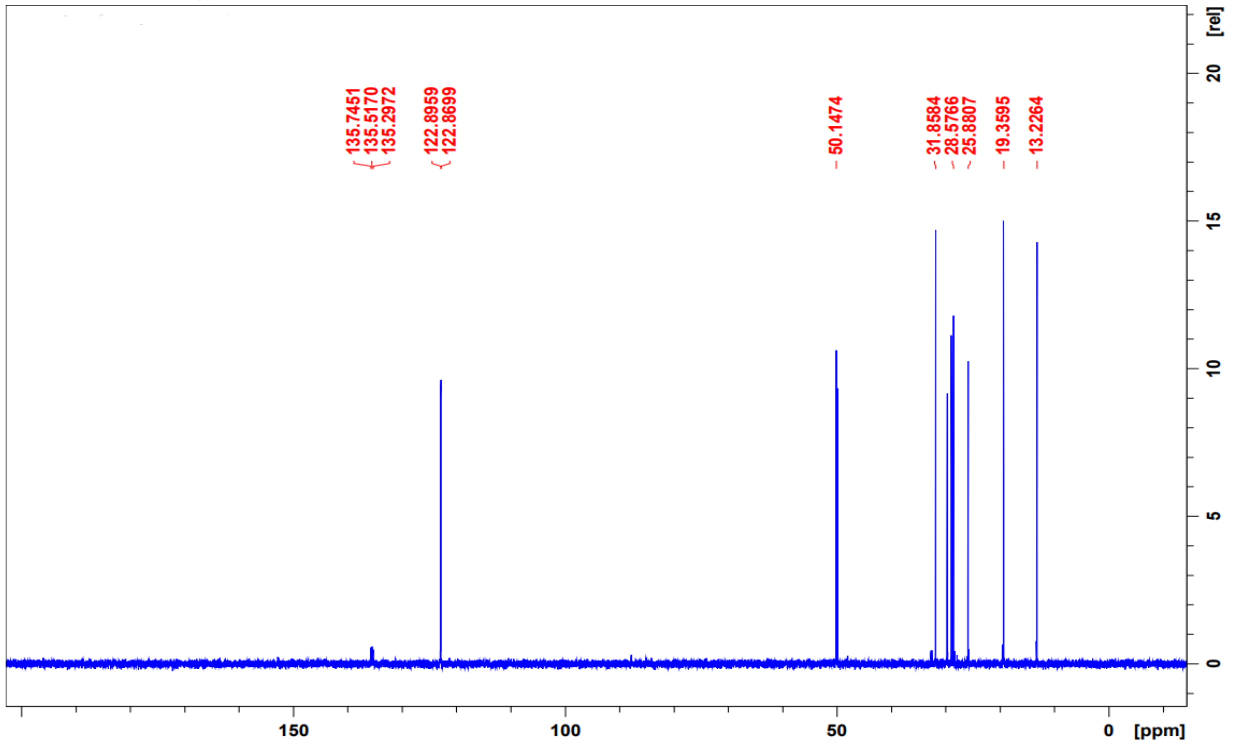

Fig.S8. ^{13}C NMR spectrum for (**4**)

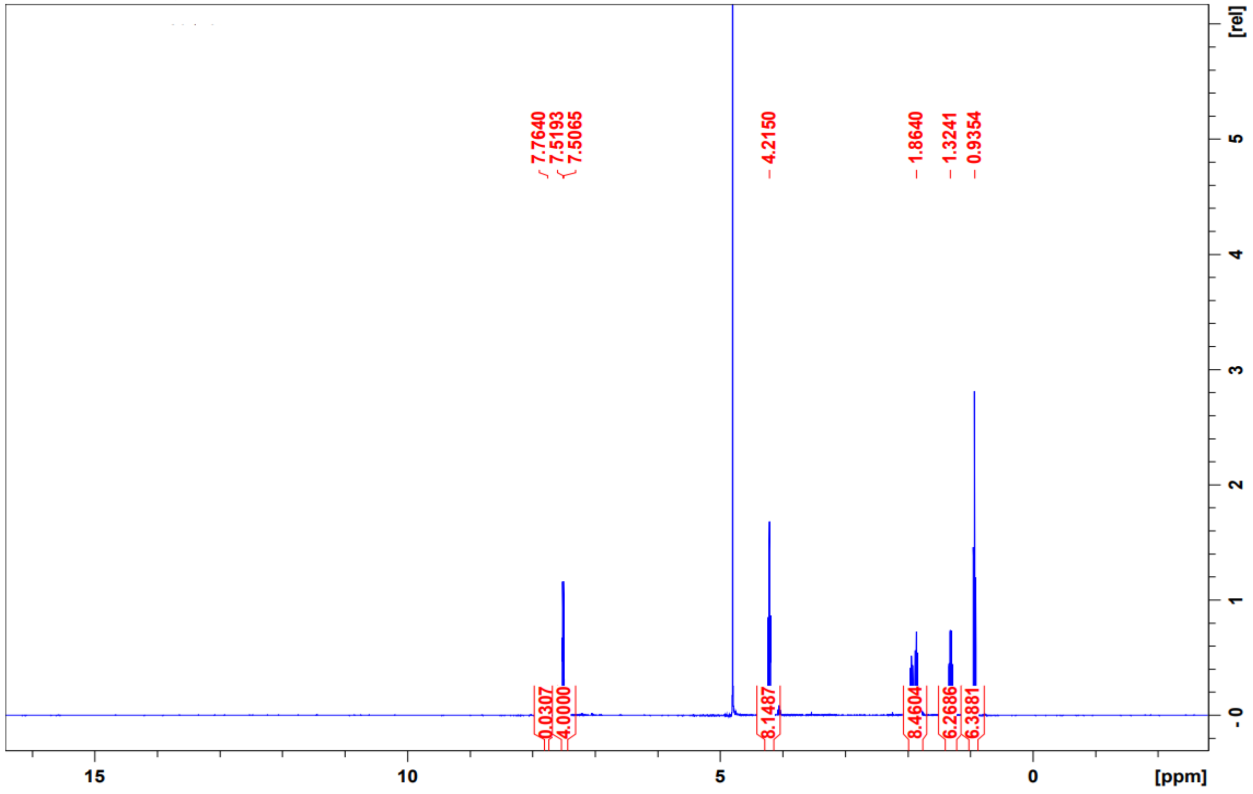

Fig.S9. ^{1}H NMR spectrum for (**5**)

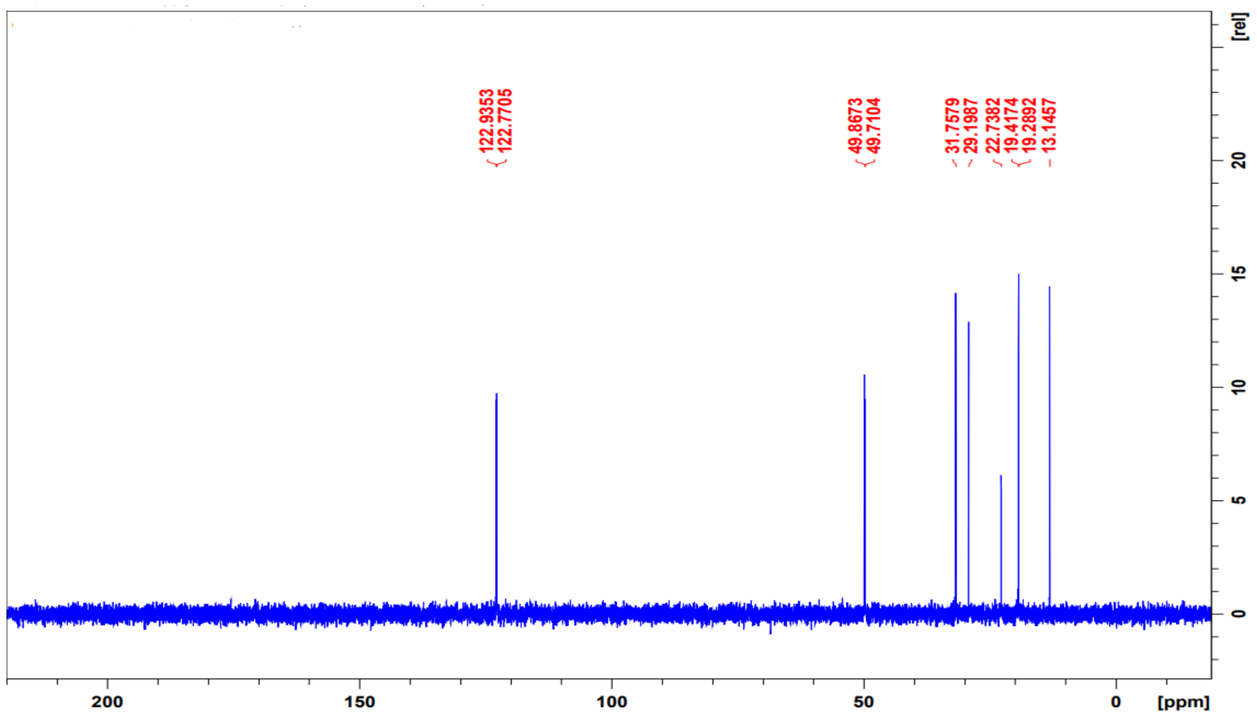

Fig.S10. ^{13}C NMR spectrum for (**5**)

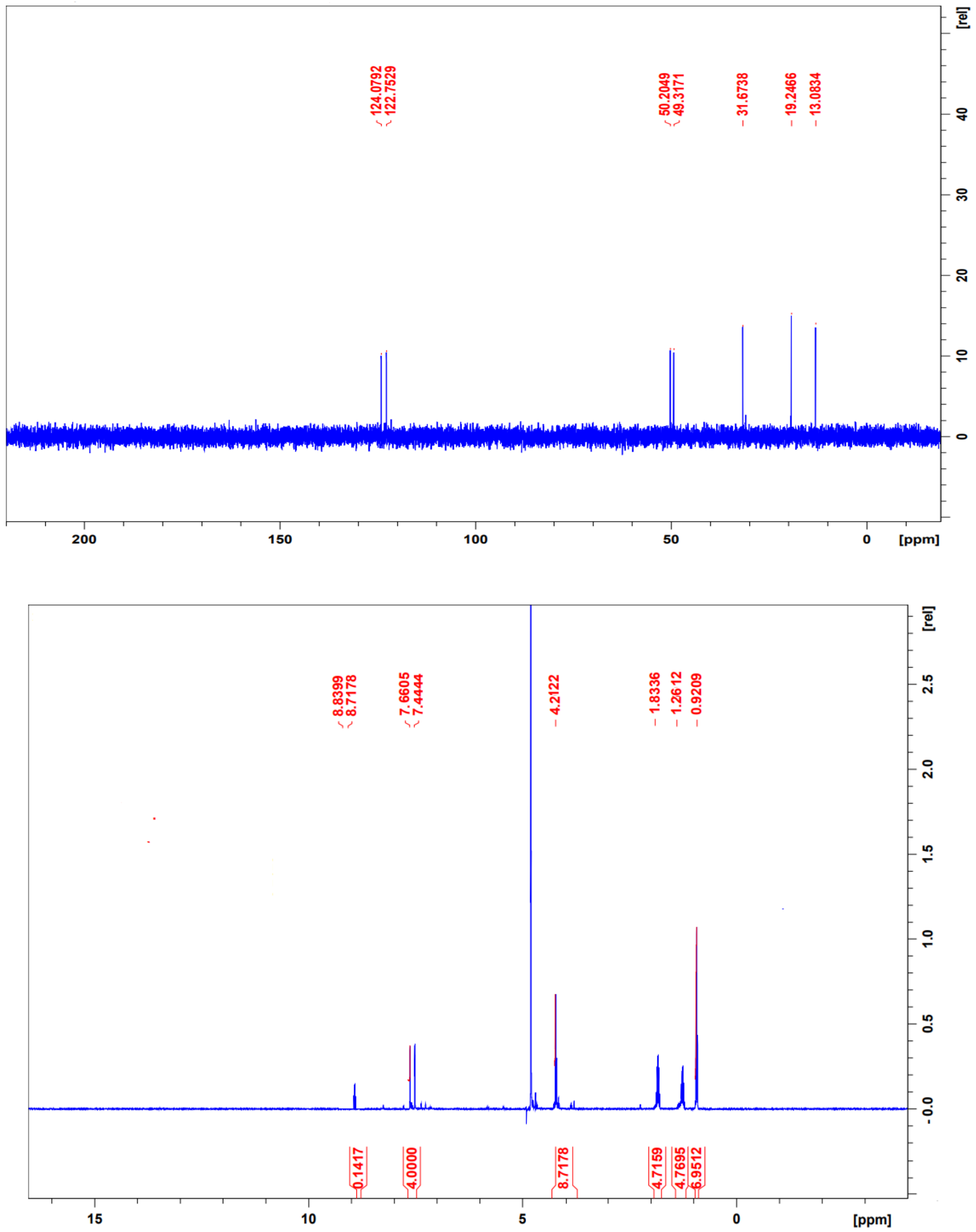

Fig.S11. ^{13}C NMR, top and ^{1}H NMR, bottom spectrum for (**6**)

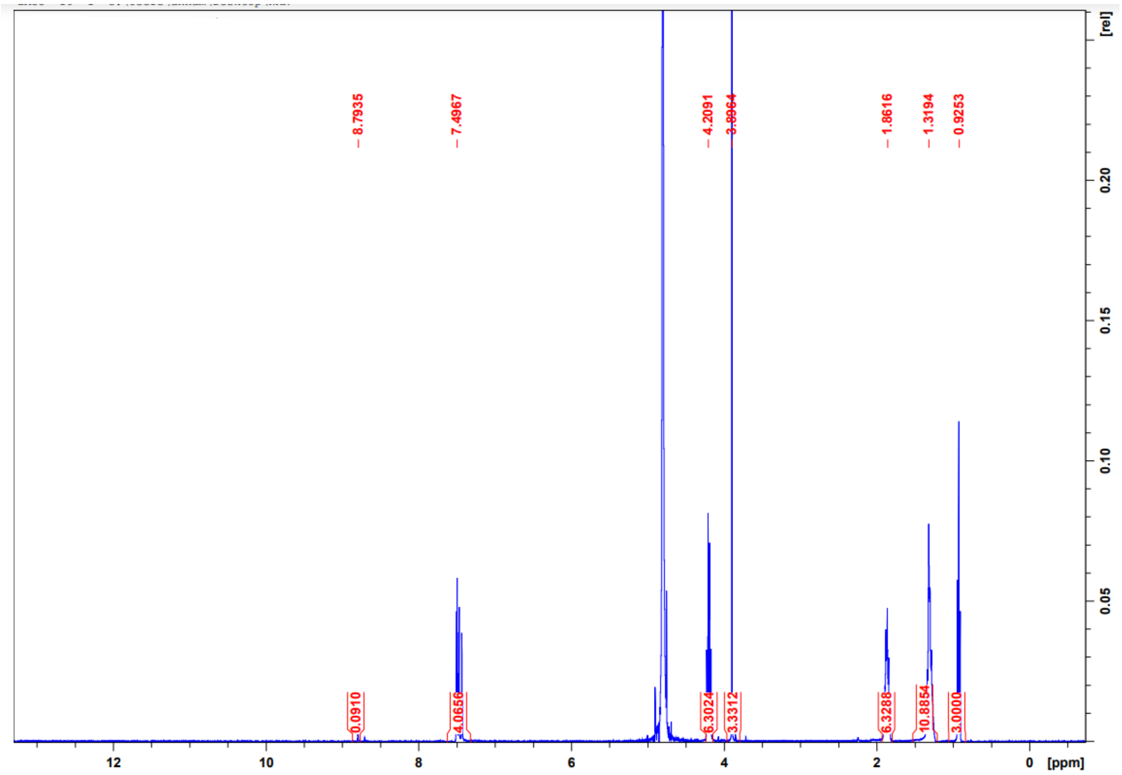

Fig.S12. ^{1}H NMR spectrum for (**7**)

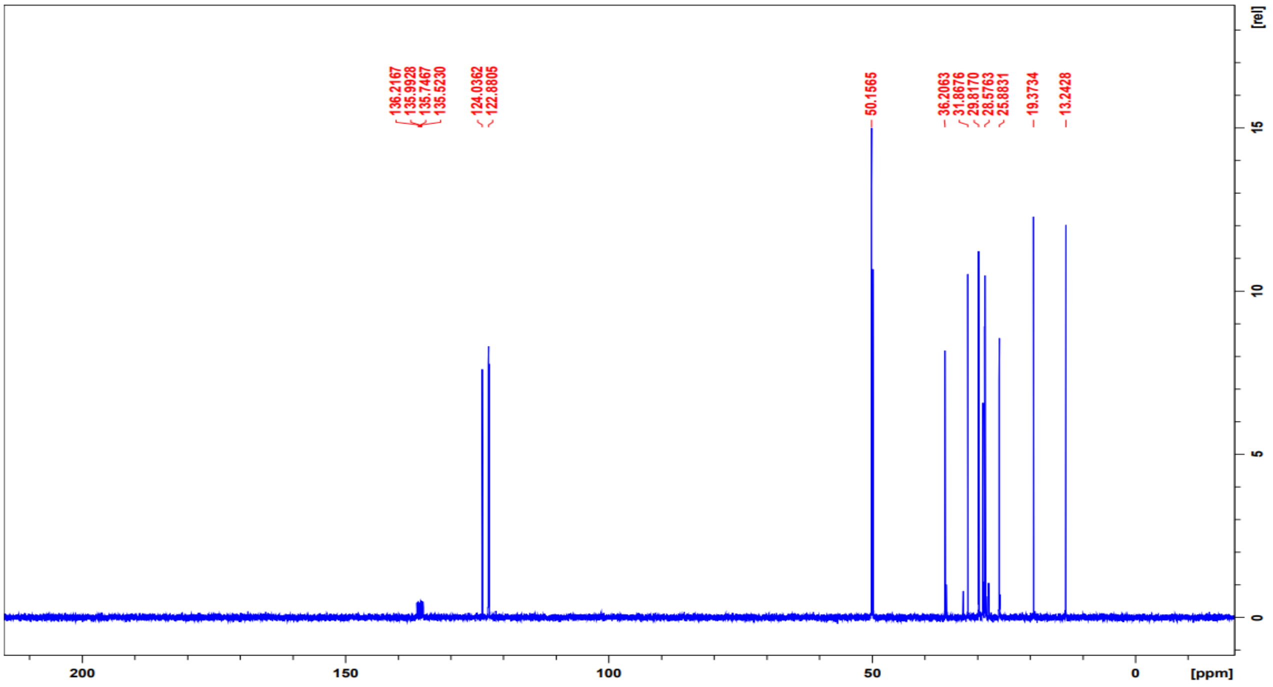

Fig.S13. ^{13}C NMR spectrum for (**7**)

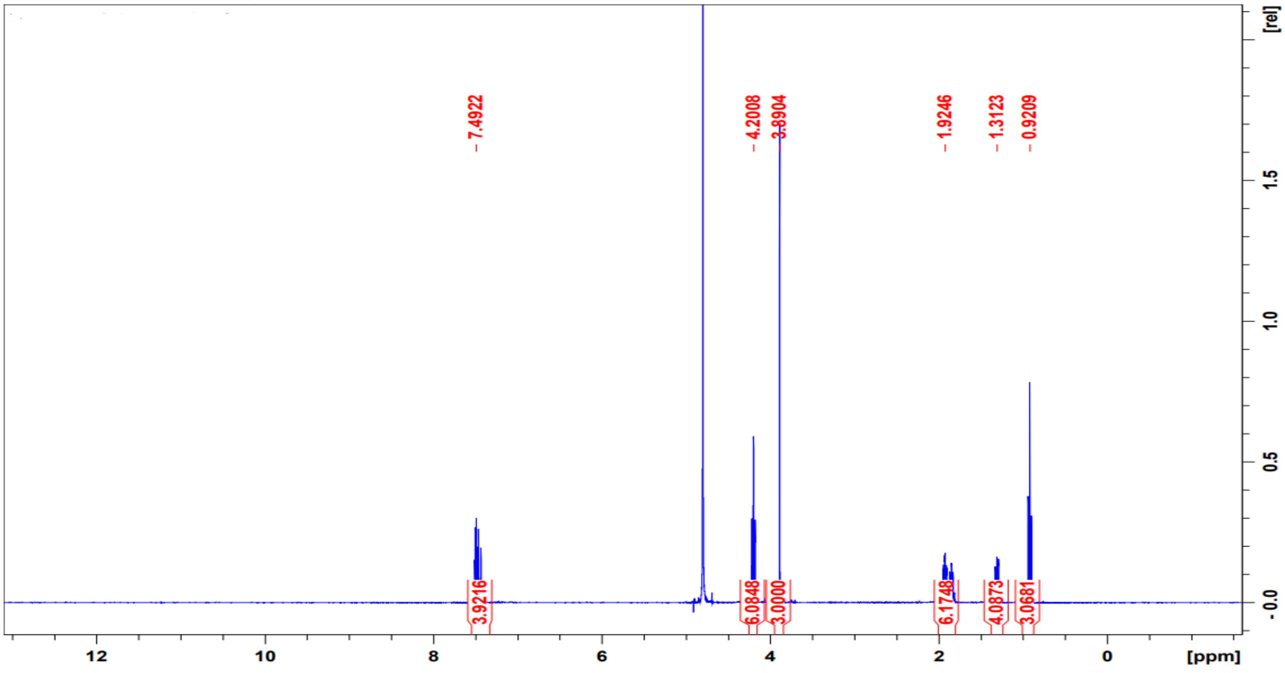

Fig.S14. ^{1}H NMR spectrum for (**8**)

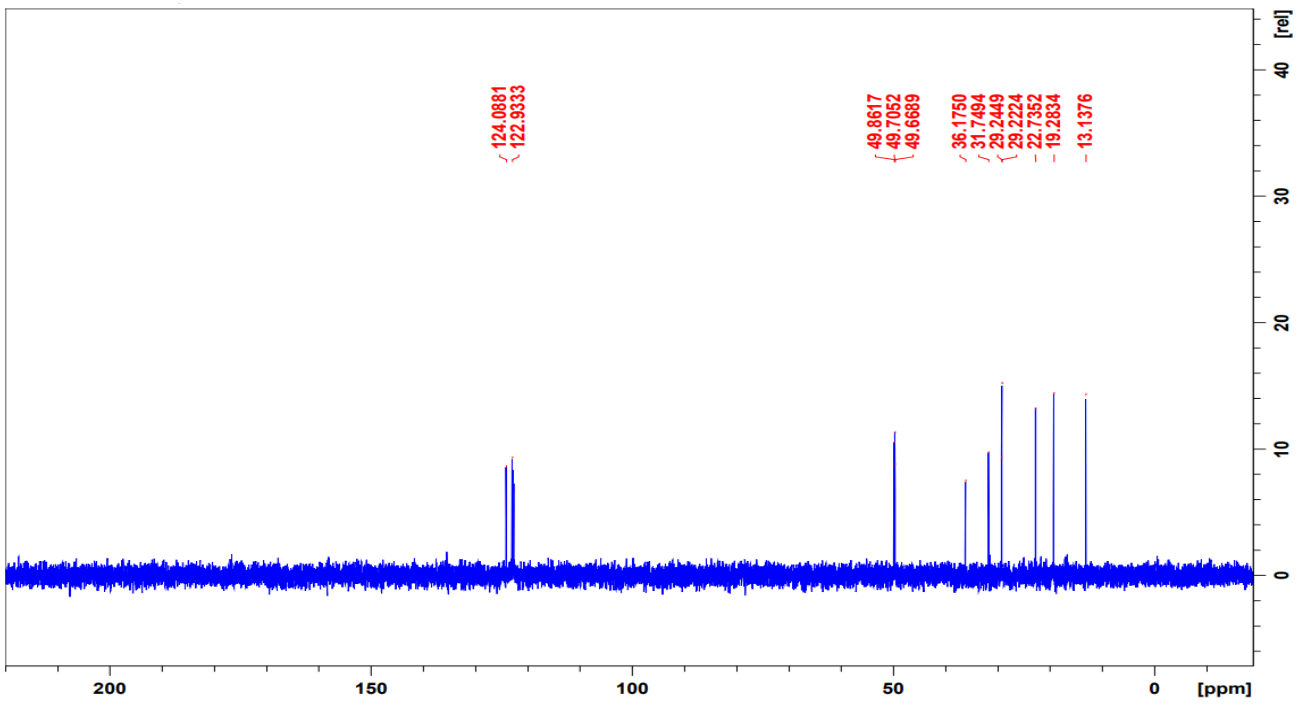

Fig.S15. ^{13}C NMR spectrum for (**8**)

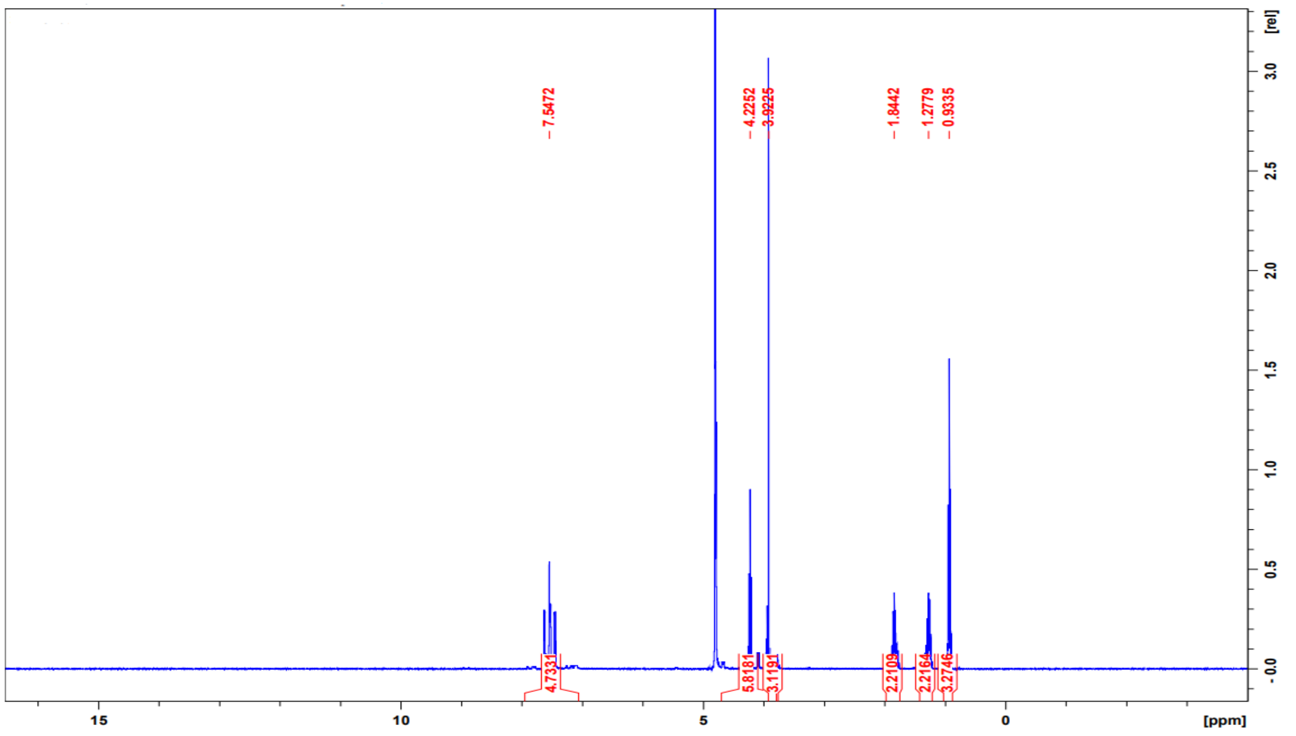

Fig.S16. ^{1}H NMR spectrum for (**9**)

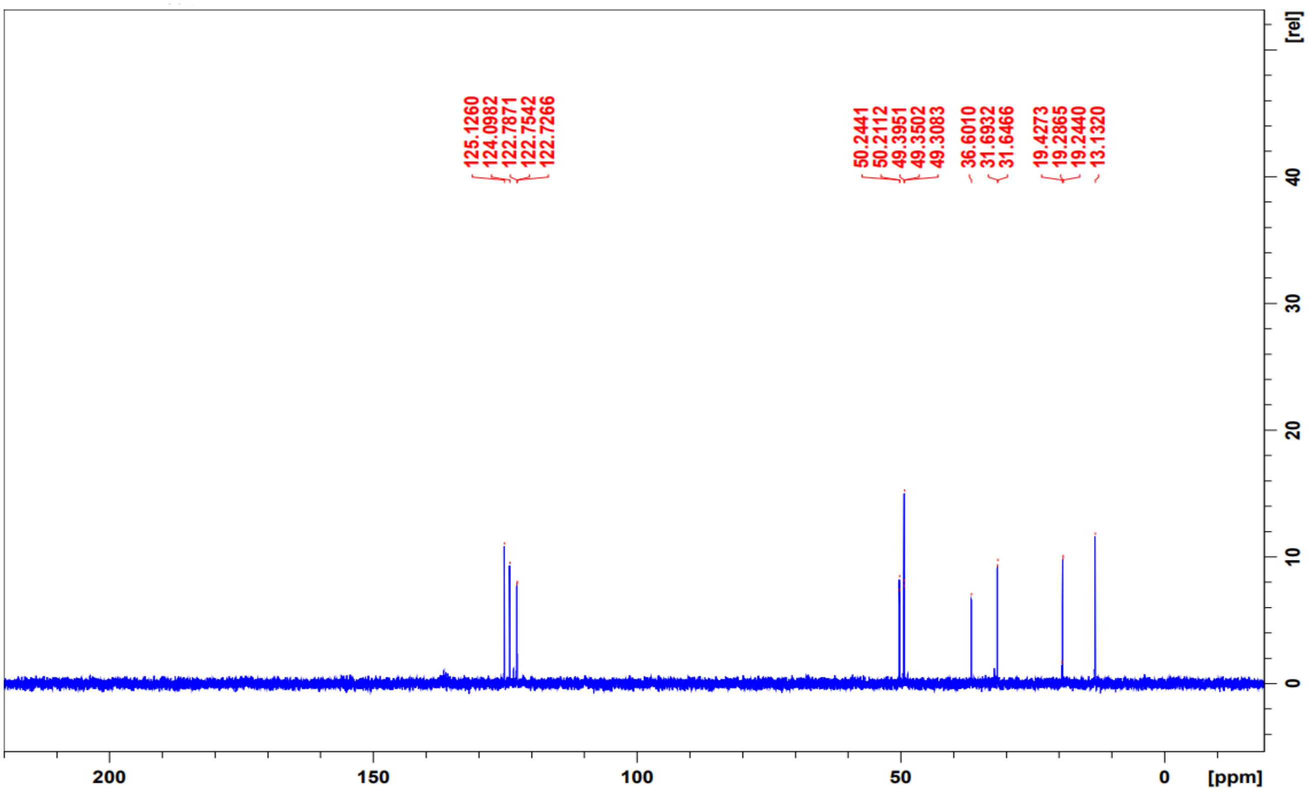

Fig.S17. ^{13}C NMR spectrum for (**9**)

2. Mass spectrometry ESI-MS (positive ion mode(+ve).

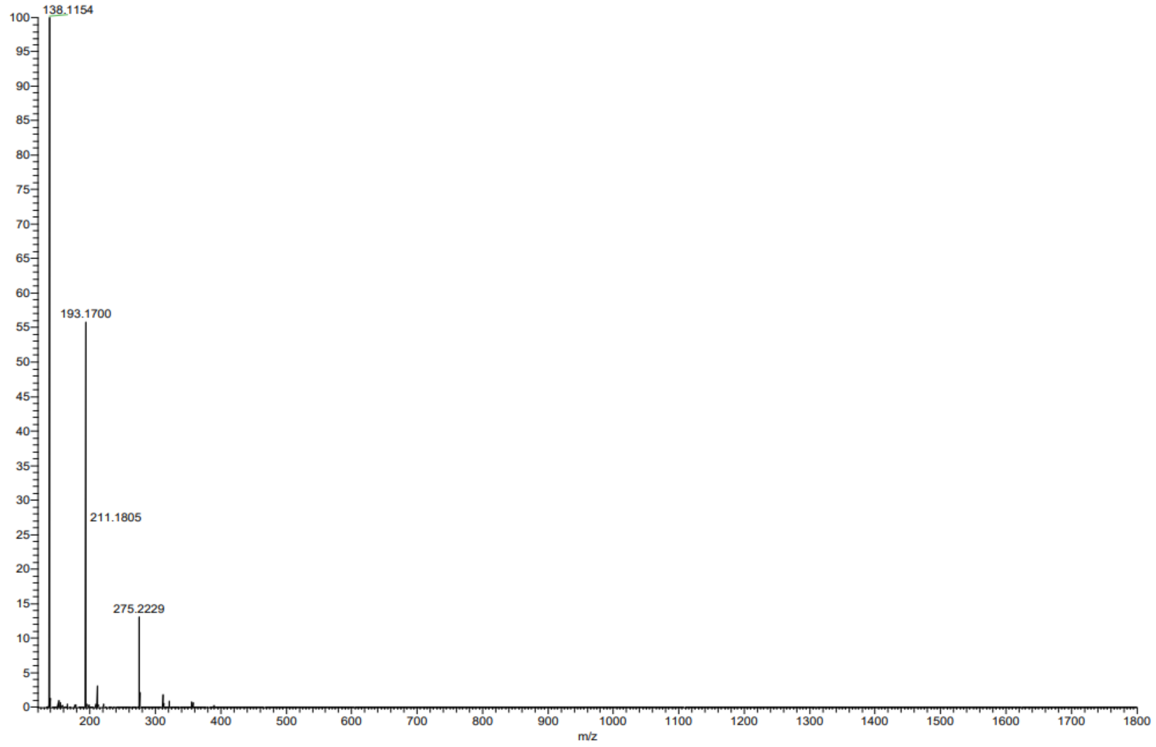

Fig.S18. ESI^+ mass spectrum for (**1**)

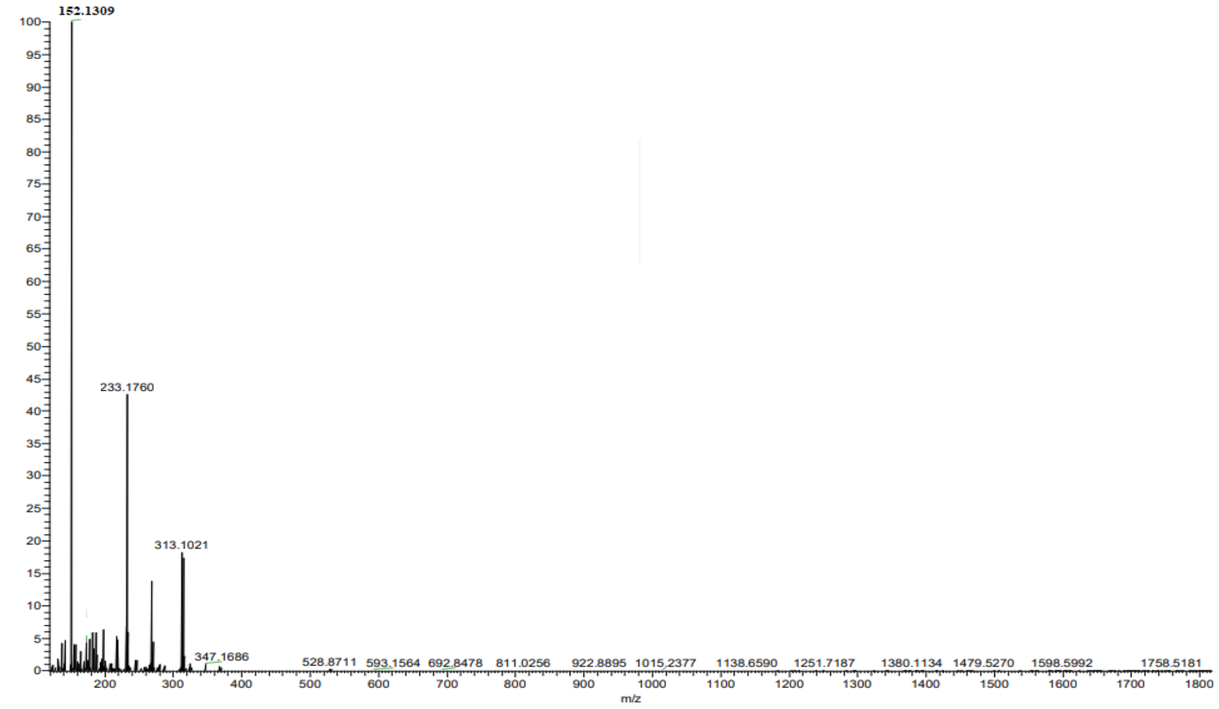

Fig.S19. ESI^+ mass spectrum for (**2**)

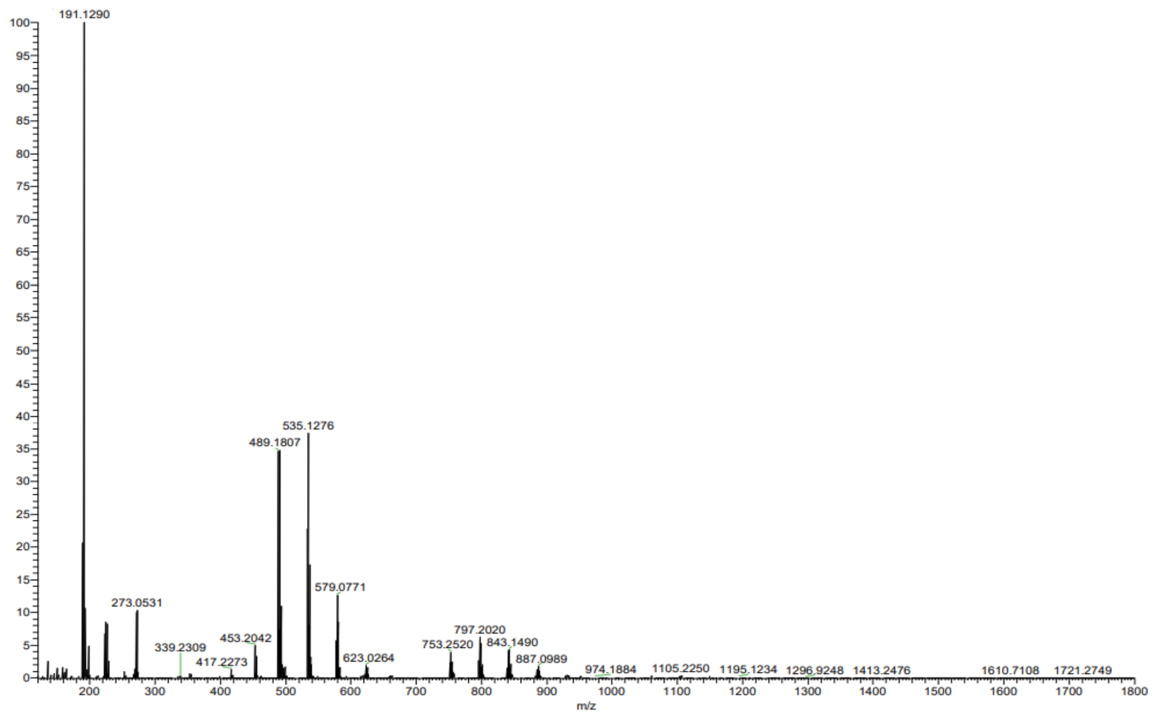

Fig.S20. ESI$^+$ mass spectrum for (**3**)

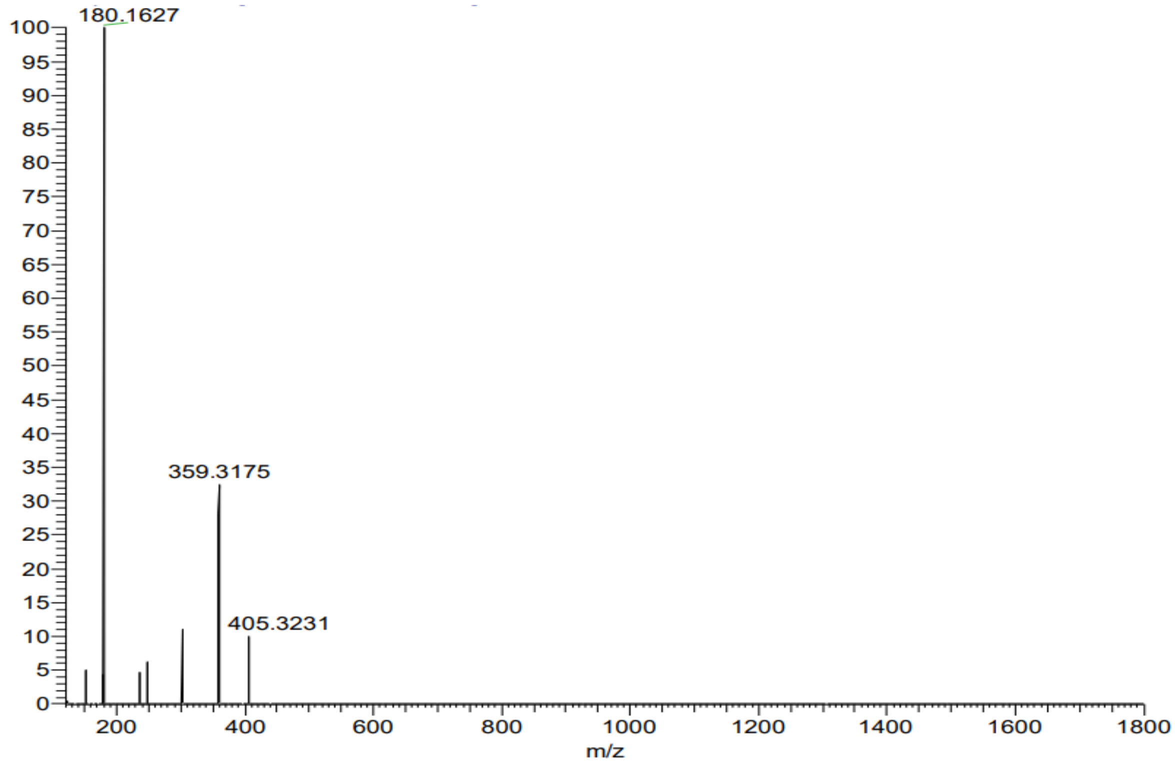

Fig.S21. ESI$^+$ mass spectrum for (**4**)

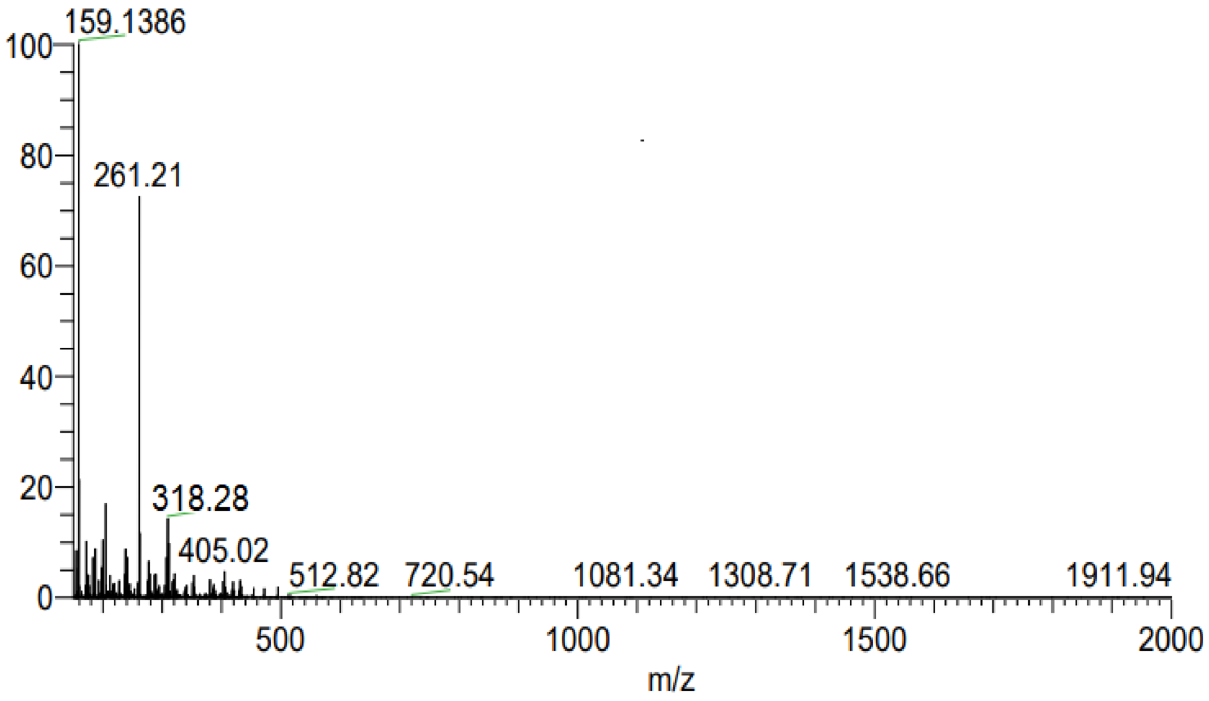

Fig.S22. ESI$^+$ mass spectrum for (**5**)

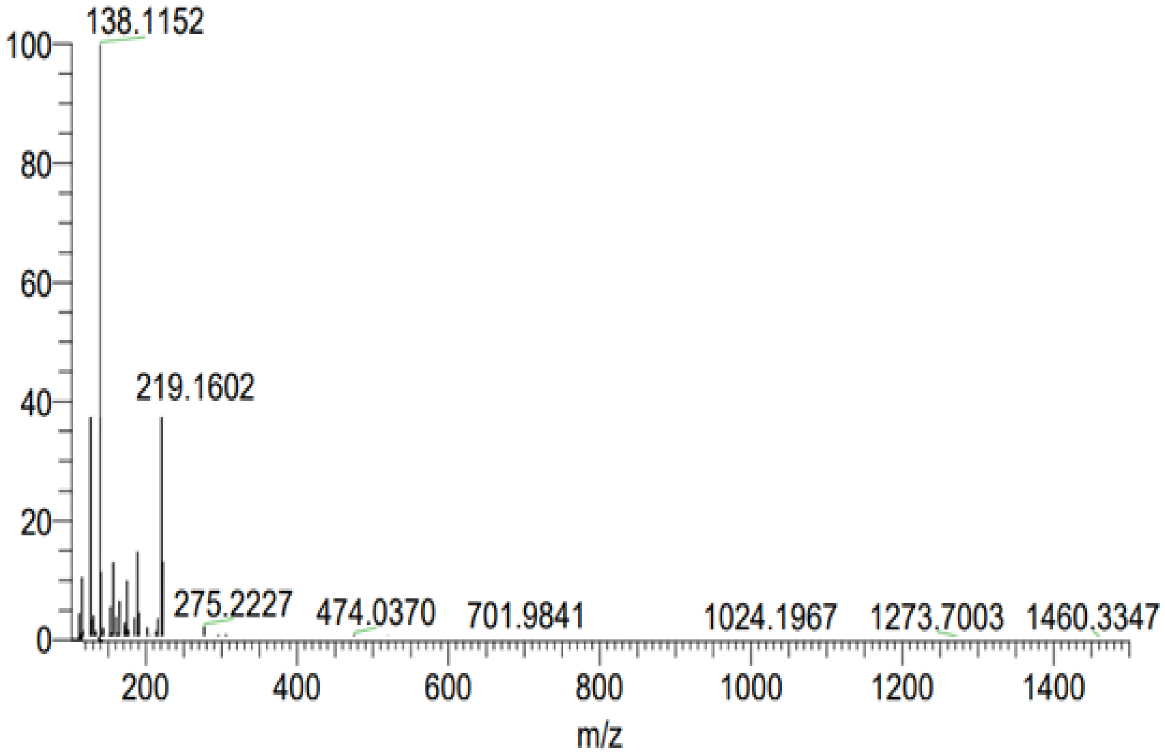

Fig.S23. ESI$^+$ mass spectrum for (**6**)

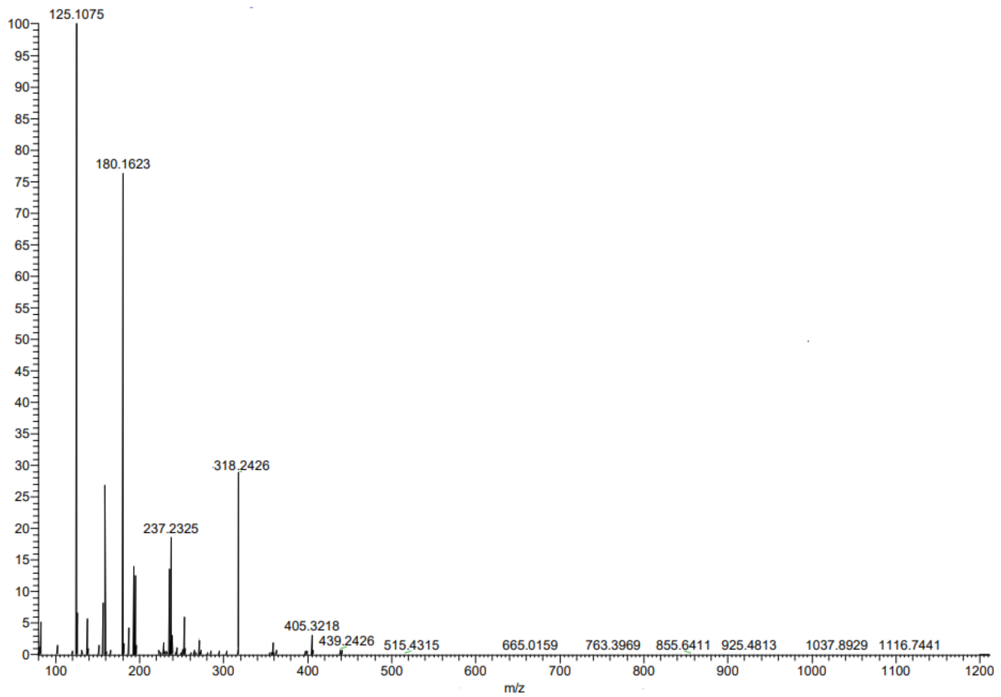

Fig.S24. ESI^{+} mass spectrum for (**7**)

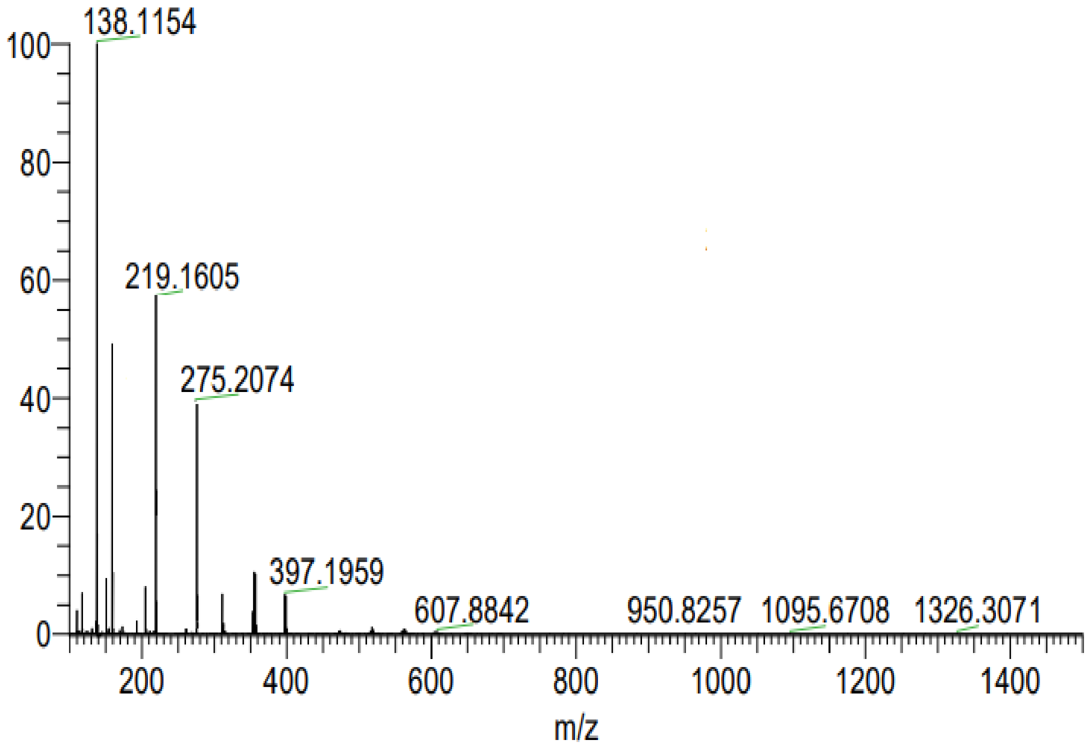

Fig.S25. ESI^{+} mass spectrum for (**8**)

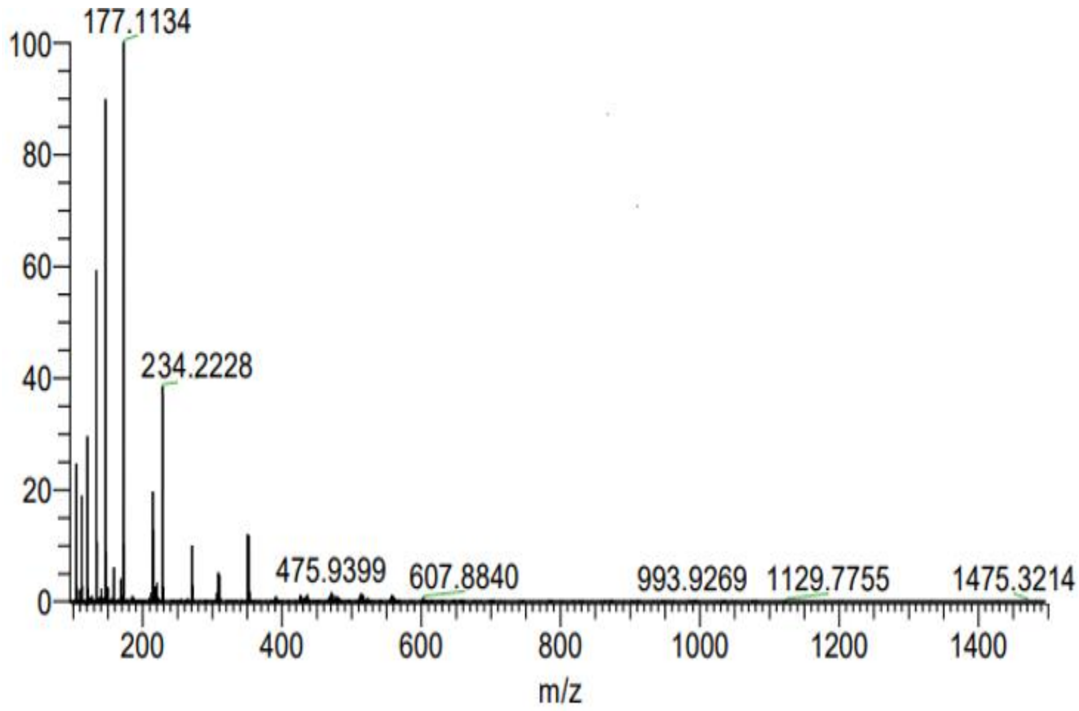

Fig.S26. ESI$^+$ mass spectrum for (**9**)

3. X-ray Crystallography

Table S1. Fractional Atomic Coordinates (×104) and Equivalent Isotropic Displacement Parameters (Å2×103) for (1). Ueq is defined as 1/3 of the trace of the orthogonalised UIJ tensor.

**Atom**	***x***	***y***	***z***	**U(eq)**
Fe1	1930.4(7)	2307.2(4)	6830.6(4)	42.0(3)
Cl1	1333(8)	2078(5)	5328(4)	101(2)
Cl2	3025(11)	849(6)	7354(5)	62.8(13)
Cl3	-470(9)	2992(6)	7463(5)	62.1(11)
Cl4	4123(6)	3467(3)	7000(4)	52.5(8)
Br1	1273(3)	2074(2)	5336.8(17)	35.1(6)
Br2	2652(12)	780(7)	7504(6)	62.7(13)
Br3	-349(11)	2769(7)	7533(6)	80(2)
Br4	3950(30)	3281(14)	7126(15)	57(4)
N1	6105(4)	2074(2)	4810(2)	43.8(7)
N2	7149(4)	875(2)	5654(2)	43.8(7)
C1	9395(7)	4804(4)	4626(4)	69.4(13)
C2	7899(6)	4166(3)	4963(4)	63.0(11)
C3	6681(6)	3767(3)	4199(3)	62.9(11)
C4	5333(6)	3050(3)	4517(3)	59.4(11)
C5	6501(5)	1796(3)	5656(2)	43.1(8)
C6	6563(6)	1299(3)	4257(3)	56.7(10)
C7	7190(6)	568(3)	4782(3)	58.2(10)
C8	7759(7)	292(4)	6459(3)	72.4(13)

Table S2. Anisotropic Displacement Parameters (Å2×103) for (1). The Anisotropic displacement factor exponent takes the form: -2π2[h2a*2U11+2hka*b*U12+…].

**Atom**	**U_{11}**	**U_{22}**	**U_{33}**	**U_{23}**	**U_{13}**	**U_{12}**
Fe1	47.8(4)	36.4(4)	42.6(4)	-9.2(2)	9.0(3)	2.1(2)
Cl1	118(4)	86(4)	100(4)	-15(3)	18(3)	3(3)
Cl2	94(4)	40.3(17)	53(2)	0.3(15)	-2.4(19)	10.4(19)
Cl3	58.3(14)	62(3)	69.5(16)	-15.7(17)	25.3(11)	8.8(15)
Cl4	56.7(13)	42.0(15)	59.9(16)	-18.1(12)	11.8(10)	-10.1(12)
Br1	41.7(12)	32.0(14)	31.5(12)	-7.8(10)	1.1(9)	-2.8(10)
Br2	84(3)	49.2(14)	54(2)	10.1(13)	3.0(15)	0.4(15)
Br3	82(3)	73(4)	92(3)	-9(2)	53(2)	11.5(19)
Br4	69(4)	48(7)	56(5)	-17(4)	23(3)	-32(3)
N1	48.3(16)	39.7(16)	44.2(16)	3.7(12)	8.7(13)	-7.5(12)
N2	48.3(15)	33.1(15)	51.2(17)	5.7(12)	12.0(13)	-9.0(12)
C1	73(3)	44(2)	91(3)	19(2)	3(2)	-7(2)
C2	70(3)	41(2)	79(3)	15(2)	8(2)	-2.2(19)
C3	68(3)	45(2)	75(3)	23(2)	8(2)	-1.9(19)
C4	57(2)	51(2)	70(3)	18(2)	2.7(19)	-0.4(19)
C5	51.3(18)	39.3(18)	40.2(18)	2.4(14)	13.2(14)	-2.9(15)
C6	75(3)	54(2)	41.8(19)	-7.0(17)	11.4(18)	-14(2)
C7	78(3)	37.5(19)	62(2)	-11.5(18)	20(2)	-7.2(18)
C8	87(3)	54(3)	77(3)	30(2)	7(3)	-4(2)

Table S3. Bond Lengths for **(1)**

**Atom**	**Atom**	**Length/Å**	**Atom**	**Atom**	**Length/Å**
Fe1	Cl1	2.257(6)	N1	C6	1.375(6)

Atom	Atom	Length/Å	Atom	Atom	Length/Å
Fe1	Cl2	2.226(8)	N2	C5	1.319(5)
Fe1	Cl3	2.301(8)	N2	C7	1.354(5)
Fe1	Cl4	2.275(5)	N2	C8	1.469(5)
Fe1	Br1	2.251(2)	C1	C1[1]	1.484(10)
Fe1	Br2	2.309(9)	C1	C2	1.533(7)
Fe1	Br3	2.175(8)	C2	C3	1.508(7)
Fe1	Br4	2.036(19)	C3	C4	1.501(6)

[1]2-X,1-Y,1-Z

Table S4. Bond Angles for **(1).**

Atom	Atom	Atom	Angle/°	Atom	Atom	Atom	Angle/°
Cl1	Fe1	Cl3	110.0(2)	C5	N1	C6	107.7(3)
Cl1	Fe1	Cl4	107.2(2)	C6	N1	C4	126.6(4)
Cl2	Fe1	Cl1	105.6(3)	C5	N2	C7	108.4(3)
Cl2	Fe1	Cl3	119.5(3)	C5	N2	C8	125.8(4)
Cl2	Fe1	Cl4	107.2(3)	C7	N2	C8	125.8(4)
Cl4	Fe1	Cl3	106.7(2)	C1[1]	C1	C2	113.0(6)
Br1	Fe1	Br2	109.4(2)	C3	C2	C1	112.7(4)
Br3	Fe1	Br1	112.1(2)	C4	C3	C2	112.8(4)
Br3	Fe1	Br2	102.5(3)	N1	C4	C3	112.4(3)
Br4	Fe1	Br1	114.3(6)	N1	C5	N2	108.6(3)
Br4	Fe1	Br2	108.2(7)	C7	C6	N1	107.4(4)
Br4	Fe1	Br3	109.7(7)	C6	C7	N2	108.0(4)
C5	N1	C4	125.7(4)				

[1]2-X,1-Y,1-Z

Table S5. Hydrogen Bonds for ZA08_0m **(1).**

D	H	A	d(D-H)/Å	d(H-A)/Å	d(D-A)/Å	D-H-A/°
C3	H3AB	Br3[1]	0.97	3.05	4.017(11)	175.0
C4	H4A	Br4	0.97	3.22	4.08(2)	148.7
C4	H4AB	Br2[2]	0.97	3.07	3.821(11)	135.6
C5	H5	Cl4	0.93	2.76	3.560(6)	145.3
C5	H5	Br4	0.93	2.80	3.60(2)	145.5
C6	H6	Cl4[2]	0.93	2.90	3.717(7)	146.8
C6	H6	Br4[2]	0.93	2.88	3.65(2)	141.3
C7	H7	Cl1[3]	0.93	2.81	3.698(8)	159.2
C8	H8C	Br2[4]	0.96	3.05	3.991(12)	168.2

[1]1+X,1/2-Y,-1/2+Z; [2]+X,1/2-Y,-1/2+Z; [3]1-X,-Y,1-Z; [4]1+X,+Y,+Z

Table S6. Torsion Angles for **(1).**

A	B	C	D	Angle/°	A	B	C	D	Angle/°
N1	C6	C7	N2	-0.6(5)	C5	N1	C6	C7	1.2(5)
C1[1]	C1	C2	C3	-179.7(5)	C5	N2	C7	C6	-0.2(5)
C1	C2	C3	C4	173.7(4)	C6	N1	C4	C3	-78.5(5)
C2	C3	C4	N1	-71.5(5)	C6	N1	C5	N2	-1.3(4)
C4	N1	C5	N2	179.5(3)	C7	N2	C5	N1	1.0(4)
C4	N1	C6	C7	-179.7(4)	C8	N2	C5	N1	179.7(4)
C5	N1	C4	C3	100.5(5)	C8	N2	C7	C6	-178.9(4)

[1]2-X,1-Y,1-Z

Table S7. Hydrogen Atom Coordinates (Å×104) and Isotropic Displacement Parameters (Å2×103) for (**1**).

**Atom**	***x***	***y***	***z***	**U(eq)**
H1A	10060.79	4399.5	4224.64	83
H1AB	8896.87	5367.09	4277.76	83
H2A	8395.97	3604.57	5312.9	76
H2AB	7228.07	4571.51	5360.98	76
H3A	6084.62	4327.52	3889.86	75
H3AB	7369.67	3425.36	3764.46	75
H4A	4771.61	3352.18	5020	71
H4AB	4436.45	2937.6	4029.76	71
H5	6348.28	2183.91	6167.95	52
H6	6450.16	1293.32	3625.98	68
H7	7592.54	-51.38	4589.52	70
H8A	7795.3	-409.69	6305.14	109
H8B	6966.5	390.15	6927.86	109
H8C	8913.89	513.13	6670.23	109

Table S8. Atomic Occupancy for **(1).**

**Atom**	***Occupancy***	**Atom**	***Occupancy***	**Atom**	***Occupancy***
Cl1	0.7	Cl2	0.7	Cl3	0.7
Cl4	0.9	Br1	0.3	Br2	0.3
Br3	0.3	Br4	0.1		

4. TGA Analysis

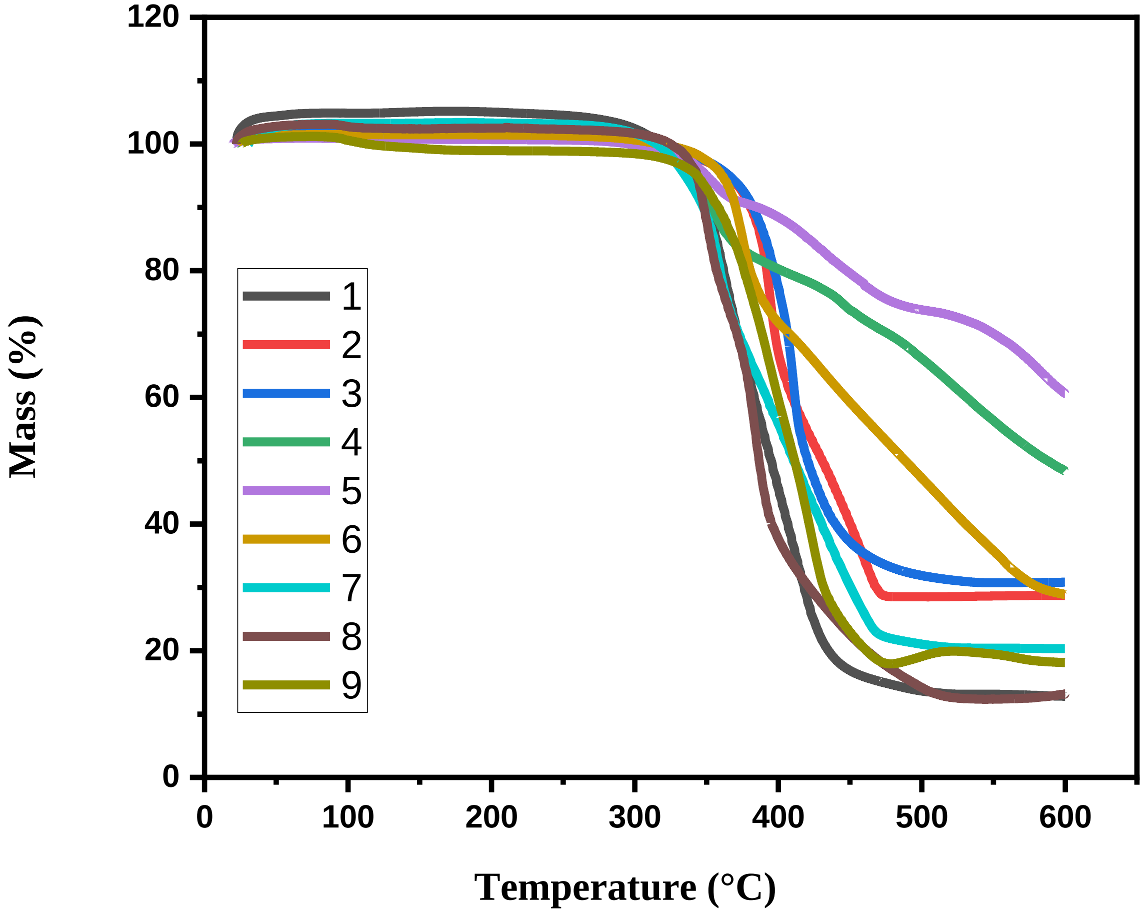

Figure S27. TGA analysis (10 °C min^{-1}, Al_2O_3 pans) of DILs (**1-9**)

5. Mass spectrometry ESI-MS (Negative ion mode(-ve).

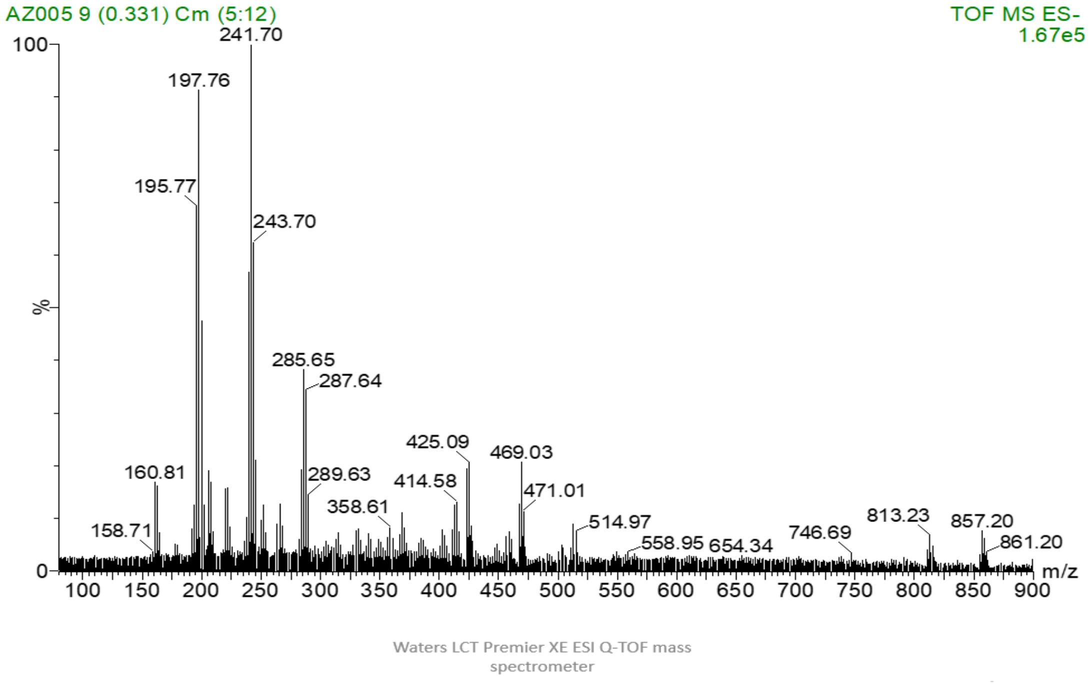

Fig.S28. ESI^{-} mass spectrum for (**5**)

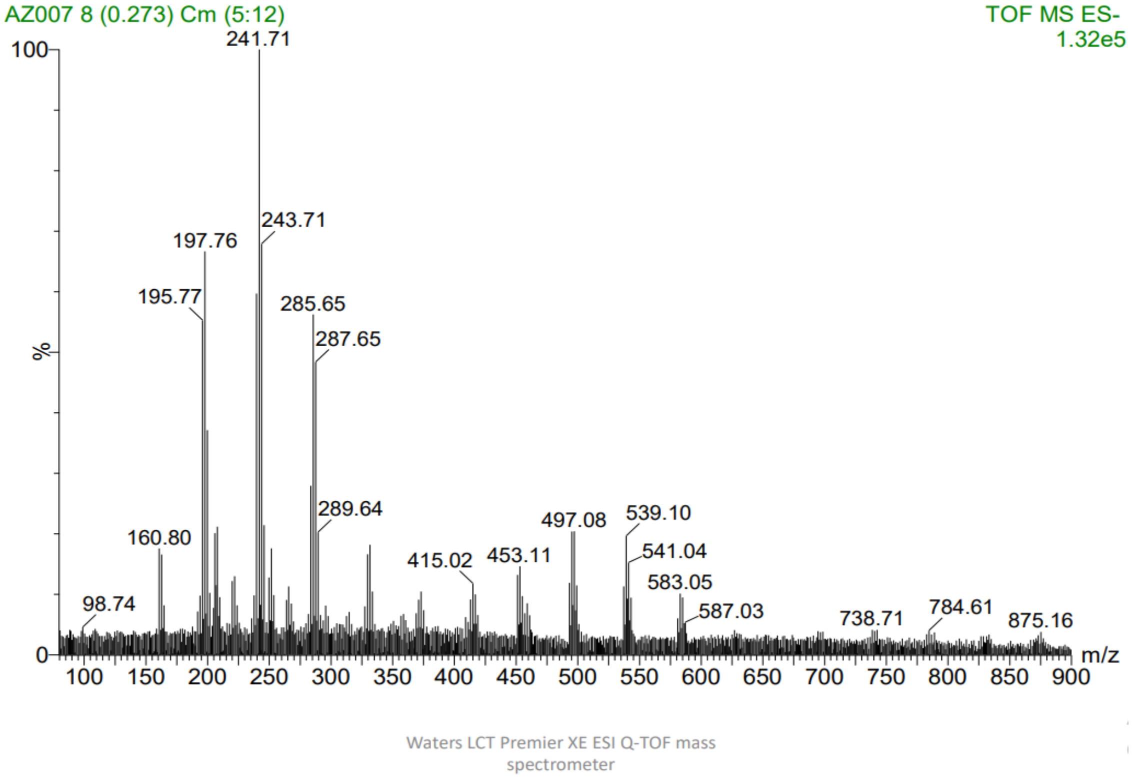

Fig.S29. ESI^{-} mass spectrum for (**7**)

6. UV-vis spectrophotometry

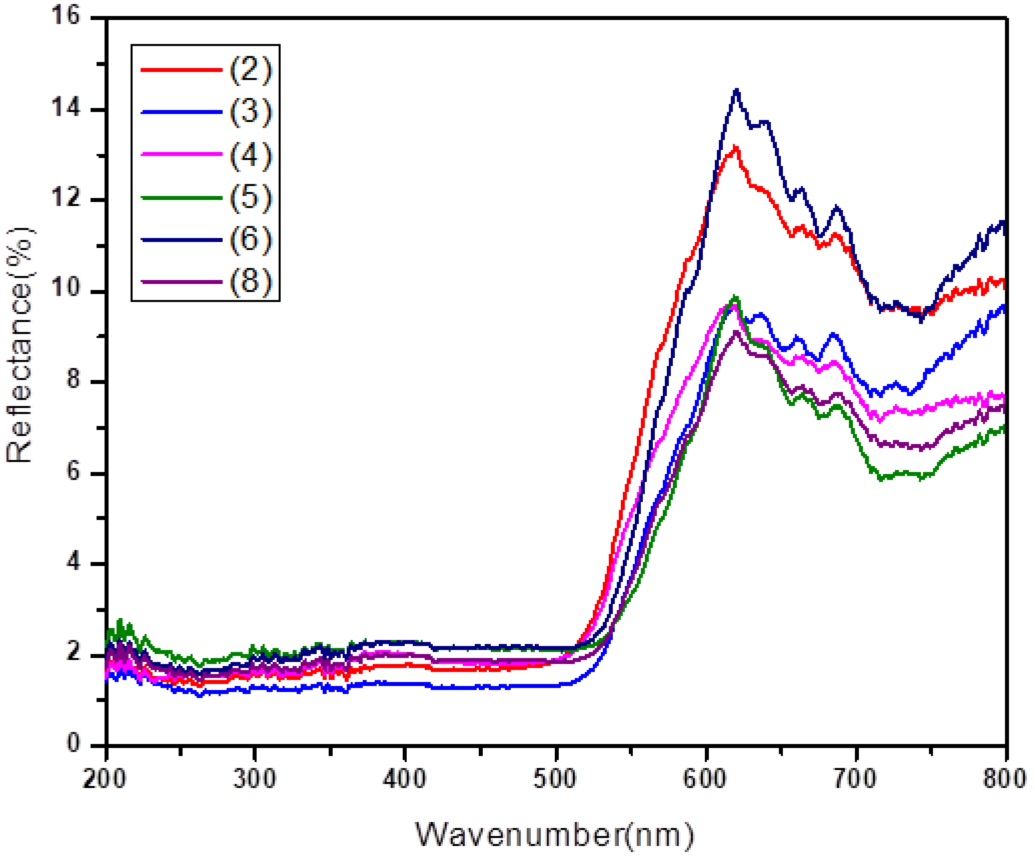

Fig.S30. UV-vis reflectance spectra for selected DcILs

7. Magnetic Properties

These Fe incorporated DcILs exhibit paramagnetic behavior over temperature range 0-300 K obeying the Curie–Weiss law in Zero-field conditions (ZFC) down to 50 K. There is a small deviation from paramagnetic behavior at the lower temperatures and follows the ferromagnetic behavior from 0-50 K as demonstrated in Figure. 9(a). and the product of molar magnetic susceptibility and temperature (**χ.T**) and χ^{-1} as a function of temperature for DcILs at 0 Gs are illustrated in 9(b)

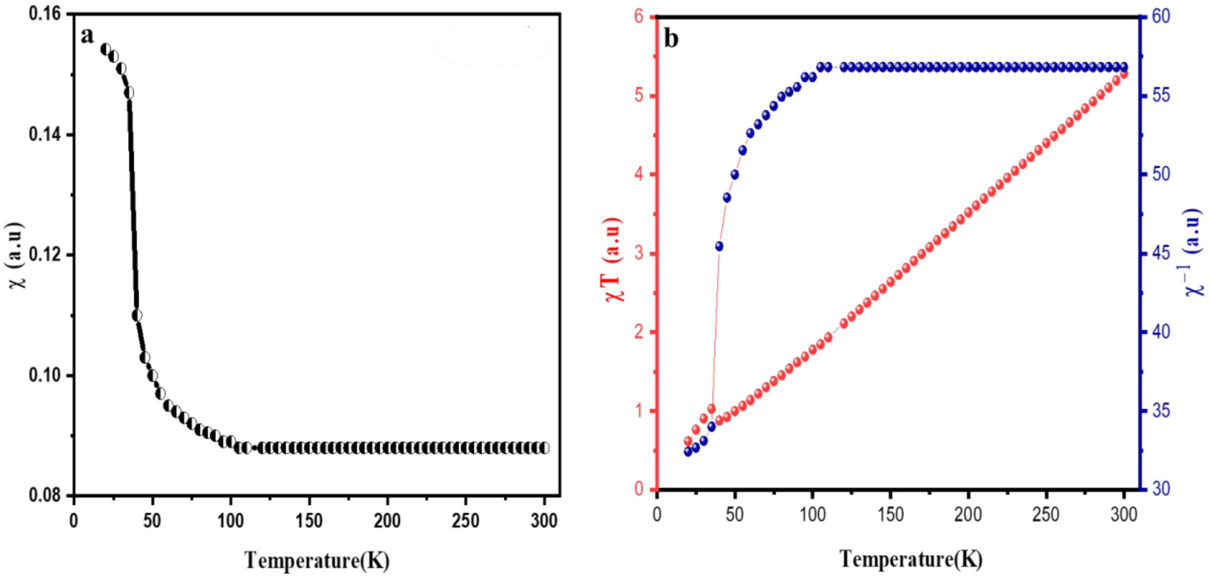

Figure S31. (a) Temperature dependent magnetization profile **(b)** The product of molar magnetic susceptibility and temperature (**χ.T**) and χ^{-1} as a function of temperature for DcILs at 0 Gs

The Ac susceptometer is used to measure the magnetization value of the DcILs under different applied magnetic fields. For DcILs with magnetic properties, no coercivity and reminiscences is anticipated on the removal of the magnetic field. The magnetic susceptibility increases on increasing the applied magnetic field and the paramagnetic linear responses of DcILs were observed at room temperature as shown in Figure 10.

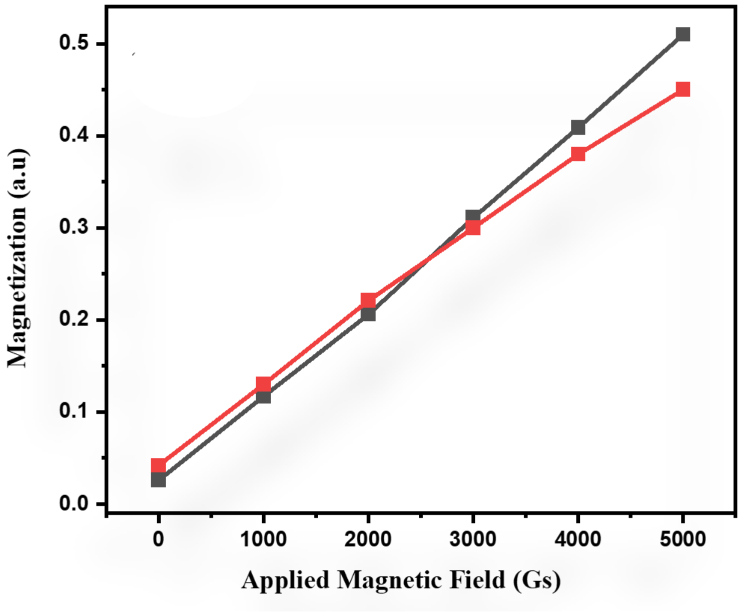

Figure S32. The relationship between magnetization of DcILs **(1&6)** and applied magnetic field

It has been observed by the magnetic studies that this type of DcILs exhibited excellent magnetic properties and were attracted towards the external magnetic field[1]. Furthermore, dications can bind very well with anions through hydrogen bonding and electrostatic interactions which not only strengthen the mutual coupling of anions and cations but also enhance the magnetic properties of metal incorporated DcILs. The strong paramagnetic properties of binuclear iron incorporated ILs are thus, favorable as a catalyst because of their easy separation on applying an external magnetic field that can greatly minimize the loss of catalyst in the extraction process.

References

1 T. Wang, W. H. Yu, T. X. Li, Y. T. Wang, J. J. Tan, B. Hu and L. H. Nie, *New J. Chem.*, **2019,** *43*, 19232–19241.
